# Longitudinal pathways of cerebrospinal fluid and positron emission tomography biomarkers of amyloid-β positivity

**DOI:** 10.1038/s41380-020-00950-w

**Published:** 2020-12-11

**Authors:** Arianna Sala, Agneta Nordberg, Elena Rodriguez-Vieitez, for the Alzheimer’s Disease Neuroimaging Initiative

**Affiliations:** 1 Division of Clinical Geriatrics, Center for Alzheimer Research, Department of Neurobiology, Care Sciences and Society, Karolinska Institutet, Stockholm, Sweden; 2 Vita-Salute San Raffaele University, Milan, Italy; 3 In Vivo Human Molecular and Structural Neuroimaging Unit, Division of Neuroscience, IRCCS San Raffaele Scientific Institute, Milan, Italy; 4 Theme Aging, The Aging Brain, Karolinska University Hospital, Stockholm, Sweden

**Keywords:** Prognostic markers, Diagnostic markers, Predictive markers

## Abstract

Mismatch between CSF and PET amyloid-β biomarkers occurs in up to ≈20% of preclinical/prodromal Alzheimer’s disease individuals. Factors underlying mismatching results remain unclear. In this study we hypothesized that CSF/PET discordance provides unique biological/clinical information. To test this hypothesis, we investigated non-demented and demented participants with CSF amyloid-β_42_ and [18F]Florbetapir PET assessments at baseline (*n* = 867) and at 2-year follow-up (*n* = 289). Longitudinal trajectories of amyloid-β positivity were tracked simultaneously for CSF and PET biomarkers. In the longitudinal cohort (*n* = 289), we found that participants with normal CSF/PET amyloid-β biomarkers progressed more frequently toward CSF/PET discordance than to full CSF/PET positivity (*χ*^2^_(1)_ = 5.40; *p* < 0.05). Progression to CSF+/PET+ status was ten times more frequent in cases with discordant biomarkers, as compared to csf−/pet− cases (*χ*^2^_(1)_ = 18.86; *p* < 0.001). Compared to the CSF+/pet− group, the csf−/PET+ group had lower *APOE*-ε4ε4 prevalence (*χ*^2^_(6)_ = 197; *p* < 0.001; *n* = 867) and slower rate of brain amyloid-β accumulation (*F*_(3,600)_ = 12.76; *p* < 0.001; *n* = 608). These results demonstrate that biomarker discordance is a typical stage in the natural history of amyloid-β accumulation, with CSF or PET becoming abnormal first and not concurrently. Therefore, biomarker discordance allows for identification of individuals with elevated risk of progression toward fully abnormal amyloid-β biomarkers, with subsequent risk of neurodegeneration and cognitive decline. Our results also suggest that there are two alternative pathways (“CSF-first” vs. “PET-first”) toward established amyloid-β pathology, characterized by different genetic profiles and rates of amyloid-β accumulation. In conclusion, CSF and PET amyloid-β biomarkers provide distinct information, with potential implications for their use as biomarkers in clinical trials.

## Introduction

The abnormal deposition of amyloid-β into plaques is considered one of the earliest neuropathological events in Alzheimer’s disease. Current research diagnostic criteria for Alzheimer’s disease promote the use of either CSF (low levels of CSF amyloid-β_42_) or PET imaging (high brain retention of amyloid-β PET tracers) as equivalent measures of amyloid-β pathology [1–4]. Amyloid-β PET and CSF amyloid-β_42_ usually provide highly concordant information, which has justified their interchangeable use in the diagnosis of Alzheimer’s disease dementia [5, 6]. However, a significant proportion of cases showing discordant CSF and PET results has been consistently reported across multiple studies [6–34] (Supplementary Table 1), reaching up to ≈20% in prevalence [15] in preclinical/prodromal disease phases (e.g., [13–15, 18, 19, 28]). To date, the interpretation of the CSF/PET discordant findings is still unclear and the longitudinal evolution of the concordant and discordant CSF/PET groups remains unknown. While it has been suggested that discordance in amyloid-β biomarkers might be related to measurement issues, due to different detection thresholds of CSF vs. PET techniques [13], recent studies have indicated that biomarker discordance might be biologically relevant, with each amyloid-β biomarker providing distinct information [15, 19, 35]. There are several reasons to hypothesize that CSF and PET biomarkers measure different aspects of amyloid-β pathology. CSF amyloid-β_42_ is a marker of soluble amyloid-β, only indirectly related to fibrillar amyloid-β in the brain [36]. Furthermore, CSF amyloid-β_42_ can be affected by variations in amyloid precursor protein and amyloid-β production, and by non-fibrillar aggregation [12]. On the other hand, amyloid-β PET is a direct measure of fibrillar amyloid-β deposition in the brain, as reported in antemortem-postmortem correlative studies [37–40].

Hence, we hypothesize that discordance in amyloid-β biomarkers provides unique information and that the existence of discordant groups may result from differences in amyloid-β processing and kinetics in the CSF vs. in the brain. In particular, while CSF amyloid-β_42_ represents an instantaneous measure of ongoing amyloid-β accumulation, reflecting the availability of soluble amyloid-β in the CSF, amyloid-β PET represents an integral measure of previously accumulated amyloid-β, providing information on the resulting fibrillar amyloid-β plaque accumulation. Thus, we expect the instantaneous CSF amyloid-β_42_ concentration to be predictive of the subsequent rate of change in amyloid-β PET standardized uptake value ratio (SUVr), but not vice-versa. To test this hypothesis, we aimed to: (a) test whether baseline CSF amyloid-β_42_ levels significantly predict future rate of change in amyloid-β PET SUVr; (b) test whether amyloid-β PET SUVr at baseline does *not* significantly predict rate of change of CSF amyloid-β_42_; (c) investigate the longitudinal evolution of CSF amyloid-β_42_ and amyloid-β PET biomarkers in a longitudinal cohort of participants stratified into four groups of concordant and discordant biomarkers (csf−/pet−, CSF+/pet−, csf−/PET+, and CSF+/PET+).

## Participants and methods

### Study design

Data used in the preparation of this article were obtained from the Alzheimer’s Disease Neuroimaging Initiative (ADNI) database (adni.loni.usc.edu). The ADNI was launched in 2003 as a public–private partnership, led by Principal Investigator Michael W. Weiner, MD. The primary goal of ADNI has been to test whether serial MRI, PET, other biological markers, and clinical and neuropsychological assessment can be combined to measure the progression of mild cognitive impairment and early Alzheimer’s disease. In the current study, we included all ADNI participants with available concurrent CSF amyloid-β_42_ and [18F]Florbetapir PET imaging measurements, obtained within a 3-month time interval. All participants gave written informed consent, as approved by local ethics committees and in accordance with the Declaration of Helsinki. For up-to-date information, see www.adni-info.org.

### Participants

Data were downloaded from the ADNI database on October 24, 2018. Our cohort consisted of *n* = 867 demented patients and non-demented participants, with available CSF amyloid-β_42_ and [18F]Florbetapir PET imaging at baseline, obtained between April 2010 and April 2014 at 57 ADNI sites, within a time interval between CSF and PET measurements of 90 days (Δ*t* = 0.08 ± 17.18 days). A total of *n* = 608 cases had a second [18F]Florbetapir PET scan performed after approximately 2 years (Δ*t* = 2.01 ± 0.68 years) and *n* = 348 cases had a third scan after 4 years (Δ*t* = 4.28 ± 0.63 years) from the baseline scan. A total of *n* = 305 had a second CSF amyloid-β_42_ measurement after ~2 years from baseline (Δ*t* = 2 ± 0.11 years).

### CSF amyloid-β_42_

Following overnight fasting, CSF samples were acquired in the morning through lumbar puncture. CSF amyloid-β_42_ measurement was performed based on the multiple xMAP Luminex platform (Luminex Corp) with INNO-BIA AlzBio3 immunoassay kit (Innogenetics) [41]. Longitudinal CSF samples belonging to the same participant were measured on the same plate. All data were derived from the UPENNBIOMK_MASTER.csv dataset.

### Amyloid-β PET

[18F]Florbetapir PET acquisition and analysis are thoroughly described elsewhere [13]. Following recommendations for longitudinal studies [42] we selected a composite reference region, including the whole cerebellum, brainstem/pons, and subcortical white matter, for SUVr estimation. A summary measure of global cortical uptake was calculated as the weighted-average uptake across regions of interest including frontal, anterior/posterior cingulate, lateral parietal, and lateral temporal lobes. All data were derived from the UCBERKELEYAV45_08_09_18.csv dataset.

### Other data

Additional baseline and longitudinal measures of neurodegeneration, tau pathology, cognition, and neuropsychiatric symptomatology were available; genetic information was also retrieved. Number of participants with additional data available, at baseline and at follow-ups, is reported in the next paragraphs.

#### FDG-PET

FDG-PET acquisition and analysis are thoroughly described elsewhere [43]. A composite index for Alzheimer’s disease-like hypometabolism was available in *n* = 857 participants (*n* = 417 at 2-year follow-up). Data were retrieved from the ADNIMERGE.csv dataset.

#### Structural MRI

T1-weighted MRI image acquisition and analysis are thoroughly described elsewhere [44]. Hippocampal, ventricles and whole-brain volume data were available in *n* = 799 participants (*n* = 614 at 1-year, *n* = 427 at 1.5-year, *n* = 335 at 2-year follow-up). Volumes of target regions were corrected for total intracranial volume. Data were retrieved from ADNIMERGE.csv dataset.

#### Other MRI measures

Presence, localization and size of infarcts were evaluated by trained physicians on T1- and T2-weighted and FLAIR MRI images in *n* = 818 participants [45]. Volume of white matter hyperintensities, indexing vascular brain injury, was quantified based on a Bayesian approach to segment high-resolution T1 and FLAIR MRI images in *n* = 816 participants [46]. Data were retrieved from the MRI_INFARCTS_11_16_15.csv and UCD_ADNI2_WMH_10_26_15.csv datasets.

#### Other CSF measures

CSF phosphorylated-tau_181_ (*n* = 867 at baseline; *n* = 307 at 2-year follow-up) and total-tau (*n* = 866 at baseline; *n* = 306 at 2-year follow-up) measurements were derived through AlzBio3 immunoassay. Data were retrieved from the UPENNBIOMK_MASTER.csv dataset. CSF amyloid-β_40_ measurements were obtained in *n* = 822 participants at baseline through 2D-UPLC-tandem mass spectrometry. Data were retrieved from the UPENNMSMSABETA.csv and UPENNMSMSABETA2.csv datasets.

#### Cognitive and neuropsychiatric measures

We took into account the following scales for cognitive evaluation: Mini-Mental State Examination (MMSE) (available in *n* = 867 participants, plus *n* = 711 at ~1-year, *n* = 665 at 2-year, *n* = 535 at 3.5-year follow-up); Alzheimer’s disease Assessment Scale-cognitive subscale (ADAS-cog11) (*n* = 867; 724; 657; 528); Rey Auditory Verbal Learning Test (RAVLT), learning subscale (*n* = 866; 722; 657; 520).

Severity of depression and sleep disorders were evaluated by means of the Geriatric Depression Scale (GDS) (*n* = 865) and Neuropsychiatric Inventory, sleep subscale (NPI-K) (*n* = 860), respectively. Data were retrieved from the ADNIMERGE.csv, GDSSCALE.csv, and NPI.csv datasets.

#### Genetic data

*APOE* genotyping was performed using DNA extracted by Cogenics from a 3 ml aliquot of whole blood (*n* = 867). We considered both the number of *APOE*-ε4 and *APOE*-ε2 alleles. Polygenic hazard score (*n* = 783) was computed based on a combination of *APOE* and 31 other genetic variants, as detailed elsewhere [47]. Data were retrieved from the APOE.csv and DESIKANLAB.csv datasets.

### Statistical analysis

#### Multivariable regression analyses

We fitted linear, quadratic, and cubic regression models, testing whether baseline CSF amyloid-β_42_ predicted the rate of change in [18F]Florbetapir PET SUVr (over 2-year and 4-year follow-up; *n* = 608 and *n* = 348 participants, respectively), and whether baseline [18F]Florbetapir PET SUVr predicted the rate of change in CSF amyloid-β_42_ (over 2-year follow-up; *n* = 305 participants). CSF amyloid-β_42_ and [18F]Florbetapir PET were included in the regression models as continuous variables. Sex, age, number of *APOE*-ε4 alleles and clinical group were entered as nuisance covariates. The assumptions of normal distribution and homoscedasticity of residuals, as well as the absence of multicollinearity between predictors, were tested and met in all regression models.

#### Classification of participants

Participants were classified as either csf−/pet−, csf−/PET+, CSF+/pet− or CSF+/PET+, based on cutoffs for abnormality of CSF amyloid-β_42_ and [18F]Florbetapir PET biomarkers. CSF amyloid-β_42_ measurements were classified as abnormal (CSF+) when <192 pg/ml. This cutoff was initially established to allow optimal discrimination between healthy controls and Alzheimer’s disease cases confirmed at autopsy [48] and later validated using a diagnosis-free data-driven approach [49]. [18F]Florbetapir PET images were classified as abnormal (PET+) when global cortical uptake was >0.79 SUVr [42]. This cutoff (optimized for longitudinal studies) was derived using linear regression from a well-validated cutoff for cross-sectional assessment (1.11 SUVr) [42], initially derived in healthy controls [50], then validated for discrimination between autopsy-confirmed healthy controls and Alzheimer’s disease patients [51], and again using mixture model analysis [35].

#### Longitudinal analyses

To investigate the simultaneous longitudinal changes in CSF amyloid-β_42_ and [18F]Florbetapir PET biomarkers, stratified by csf−/pet−, csf−/PET+, CSF+/pet− or CSF+/PET+ subgroups, we selected all participants (*n* = 289) with CSF and PET amyloid-β biomarkers available both at baseline and at the 2-year follow-up examinations, and obtained within 90 days. Differences in prevalence of longitudinal outcomes across subgroups were tested by means of *χ*^2^ tests.

#### Group comparisons

Group comparisons for continuous variables were performed via general linear modeling. Post-hoc tests were performed using Bonferroni correction for multiple comparisons. The assumptions of normality of residuals and homoscedasticity were tested; when not met, the estimates were bootstrapped with 1000 replicates, generating bias-corrected and accelerated 95% confidence intervals. For categorical variables, *χ*^2^ tests were applied. Cox regression analysis was used for estimation of hazard risks. When the assumption of proportionality of hazards was not met, we run a stratified model. Sex, age, number of *APOE*-ε4 alleles, and clinical group were included as nuisance covariates.

All statistical analyses were performed in PASW Statistics 18 (SPSS Inc.), setting the significance level at *p* < 0.05 (two tailed). Graphical renderings were performed in RStudio (v.1.1.456, http://www.rstudio.com/), using ggplot2 v.2.2.1.

## Results

### Longitudinal changes in amyloid-β CSF and PET measures in the whole cohort

Table 1 shows baseline demographic and clinical characteristics of the participants, divided by clinical group (*n* = 867).

**Table 1:** Baseline descriptive statistics of the study cohort.

	Healthy controls	Subjective memory complaints	Mild cognitive impairment	Alzheimer’s disease dementia	Test value; *P* value^1^
*N* (%)	185 (21.34)	90 (10.38)	445 (51.33)	147 (16.96)	–
Sex, *N* (%) male/female	89/96 (48.11/51.89)	36/54 (40/60)	247/198 (55.51/44.49)	88/59 (59.86/40.14)	*χ*^2^_(3)_ = 11.79; *p* < 0.01
Age, years	74.74 ± 6.70 [74.31 ± 0.53]	72.15 ± 5.42 [71.98 ± 0.75]	72.06 ± 7.41 [72.11 ± 0.33]	74.66 ± 7.29 [75.15 ± 0.59]	*F*_(3,861)_ = 9.67; *p* < 0.001^a^
Education, years	16.11 ± 2.67 [16.16 ± 0.20]	16.00 ± 2.61 [16.05 ± 0.28]	16.30 ± 2.63 [16.30 ± 0.13]	16.50 ± 2.58 [16.41 ± 0.22]	*F*_(3,860)_ = 0.43; *p* = 0.73
MMSE	29.05 ± 1.16 [29.02 ± 0.09]	28.97 ± 1.26 [28.85 ± 0.13] *29 (28–30)*	28.03 ± 1.76 [28.01 ± 0.08]	22.99 ± 2.49 [23.17 ± 0.25] *23 (21–25)*	*F*_(3,860)_ = 356.18; *p* < 0.001^b^
APOE-ε4 alleles, *N* (%) (0/1/2)	138/42/5 (74.60/22.70/2.70)	61/28/1 (67.78/31.11/1.11)	229/171/45 (51.46/38.43/10.11)	51/67/29 (34.69/45.58/19.73)	*χ*^2^_(6)_ = 74.68; *p* < 0.001
APOE-ε2 alleles, *N* (%) (0/1/2)	160/25/0 (86.49/13.51/0)	78/12/0 (86.67/13.33/0)	409/36/0 (91.91/8.09/0)	140/6/1 (95.24/4.08/0.68)	*χ*^2^_(6)_ = 16.11; *p* < 0.05
CSF amyloid-β_42_, pg/ml	199.32 ± 51.88 [193.00 ± 3.28]	202.7 ± 48.5 [193.70 ± 4.65]	174.55 ± 52.32 [174.23 ± 2.08]	138.85 ± 40.06 [153.33 ± 3.71] *132 (115–151)*	*F*_(3,860)_ = 24.77; *p* < 0.001^b^
[18F]Florbetapir PET SUVr	0.80 ± 0.11 [0.81 ± 0.01]	0.82 ± 0.12 [0.84 ± 0.01]	0.88 ± 0.14 [0.88 ± 0.01]	1.01 ± 0.14 [0.99 ± 0.01]	*F*_(3,860)_ = 56.82; *p* < 0.001^b^

Results from GLM are corrected for sex, age, and number of APOE-ε4 alleles. Data are reported as mean ± standard deviation, unless indicated otherwise. Adjusted estimates of the mean and the respective standard error are reported in square brackets. Sex, age, number of APOE-ε4 alleles, and clinical group were entered as nuisance covariates. For groups where variables are non-normally distributed, median (interquartile range) is also reported, in italics.

*MMSE* Mini-Mental State Examination, *SUVr* standardized uptake value ratio.

^1^Results of post-hoc comparisons, Bonferroni-corrected for multiple comparisons; significant differences:

^a^Healthy controls vs. mild cognitive impairment; subjective cognitive complaints vs. Alzheimer’s disease dementia; mild cognitive impairment vs. Alzheimer’s disease dementia.

^b^Healthy controls vs. mild cognitive impairment; healthy controls vs. Alzheimer’s disease dementia; subjective cognitive complaints vs. mild cognitive impairment; subjective cognitive complaints vs. Alzheimer’s disease dementia; mild cognitive impairment vs. Alzheimer’s disease dementia.

**Fig. 1:**
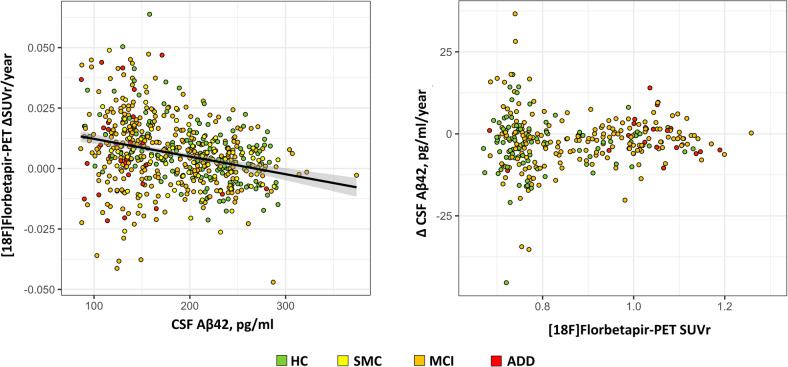
Hypothesis validation—continuous measures. Scatter plots show the association between baseline CSF amyloid-β_42_ vs. longitudinal changes in [18F]Florbetapir PET SUVr (left panel) and vice-versa (right panel). Baseline CSF amyloid-β_42_ significantly predicts the rate of change in [18F]Florbetapir PET SUVr, as computed on a 2-year time interval (left panel), the association being represented by a linear regression model. [18F]Florbetapir PET SUVr does not predict the rate of change in CSF amyloid-β_42_, as computed on a 2-year time interval (right panel). Aβ42 amyloid-β_42,_ ADD Alzheimer’s disease dementia, HC healthy control, MCI mild cognitive impairment, SMC subjective memory complaints, SUVr standardized uptake value ratio.

Testing of our hypothesis by means of standard regression models in the whole cohort, considering participants with longitudinal amyloid-β CSF (*n* = 305) and PET (*n* = 608; *n* = 348) measurements, confirmed both our predictions, i.e.,: (i) baseline CSF amyloid-β_42_ values significantly predicted the rate of change in [18F]Florbetapir PET SUVr, as computed over a 2-year (*F*_(5,602)_ = 12.11, *p* < 0.001; *n* = 608; Fig. 1) and 4-year (*F*_(5,342)_ = 15.11, *p* < 0.001; *n* = 348; data not shown) time interval. Thus, CSF amyloid-β_42_ levels linearly predicted [18F]Florbetapir PET accumulation, with lower CSF amyloid-β_42_ levels associated with faster brain [18F]Florbetapir PET accumulation. Quadratic and cubic terms did not reach statistical significance in the respective models. (ii) Neither linear, quadratic nor cubic regression models significantly predicted the 2-year rate of change in CSF amyloid-β_42_ values as a function of baseline [18F]Florbetapir PET SUVr (*F*_(4,302)_ = 1.06, *p* = 0.374; *F*_(5,301)_ = 0.92, *p* = 0.471; *F*_(6,300)_ = 0.95, *p* = 0.462, respectively; *n* = 305; Fig. 1).

### Longitudinal changes in amyloid-β CSF and PET measures for each of the CSF/PET subgroups

Table 2 shows baseline demographic and clinical characteristics of the participants, divided by CSF amyloid-β_42_ and [18F]Florbetapir PET biomarker profiles (*n* = 867).

**Table 2:** Baseline descriptive statistics for concordant and discordant biomarker groups.

	csf−/pet−	csf−/PET+	CSF+/pet−	CSF+/PET+	Test value; *p* value^1^
*N* (%)	300 (34.60)	44 (5.07)	62 (7.15)	461 (53.17)	–
Clinical group, *N* (%) (healthy controls/subjective memory complaints/mild cognitive impairment/Alzheimer’s disease dementia)	99/43/145/13 (33/14.33/48.33/4.33)	9/12/22/1 (20.45/27.27/50/2.27)	23/6/28/5 (37.10/9.68/45.16/8.06)	54/29/250/128 (11.71/6.29/54.23/27.77)	*χ*^2^_(9)_ = 140.64; *p* < 0.001
Sex, *N* (%) (male/female)	164/136 (54.67/45.33)	14/30 (31.82/68.18)	42/20 (67.74/32.26)	240/221 (52.06/47.94)	*χ*^2^_(3)_ = 13.83; *p* < 0.005
Age, years	72.00 ± 7.36 [70.41 ± 0.44]	70.25 ± 6.35 [69.67 ± 0.91]	74.22 ± 7.84 [74.31 ± 1.13]	73.90 ± 7.10 [75.19 ± 0.37]	*F*_(3,860)_ = 21.04; *p* < 0.001^a^
Education, years	16.21 ± 2.63 [16.37 ± 0.17]	16.11 ± 2.53 [16.23 ± 0.40]	15.82 ± 2.65 [15.87 ± 0.34]	16.37 ± 2.63 [16.25 ± 0.14]	*F*_(3,859)_ = 0.6; *p* = 0.613
MMSE	28.57 ± 1.70 [27.79 ± 0.12] *29 (28–30)*	28.64 ± 1.82 [27.94 ± 0.29] *29 (28–30)*	28.18 ± 2.18 [27.66 ± 0.25]	26.59 ± 3.06 [27.23 ± 0.12]	*F*_(3,859)_ = 3.51; *p* < 0.05^b^
APOE-ε4 alleles, *N* (%) (0/1/2)	251/49/0 (83.67/16.33/0)	30/14/0 (68.18/31.82/0)	40/15/7 (64.52/24.19/11.29)	158/230/73 (34.27/49.89/15.84)	*χ*^2^_(6)_ = 197; *p* < 0.001
APOE-ε2 alleles, *N* (%) (0/1/2)	254/46/0 (84.67/15.33/0)	38/6/0 (86.36/13.64/0)	55/7/0 (88.71/11.29/0)	440/20/1 (95.44/4.34/0.22)	*χ*^2^_(6)_ = 29.93; *p* < 0.001
CSF amyloid-β_42_, pg/ml	235.41 ± 27.94 [230.18 ± 1.58]	231.57 ± 28.83 [227.03 ± 3.77]	161.94 ± 23.80 [160.91 ± 3.16]	135.26 ± 23.69 [139.24 ± 1.26]	*F*_(3,859)_ = 625.34; *p* < 0.001^c^
[18F]Florbetapir PET SUVr	0.73 ± 0.03 [0.75 ± 2.9E−03]	0.82 ± 0.03 [0.83 ± 5.88E−03] *0.81 (0.80–0.83)*	0.75 ± 0.03 [0.76 ± 4.41E−03]	1 ± 0.1 [0.99 ± 5E−03]	*F*_(3,859)_ = 487.61; *p* < 0.001^d^

Results from GLM are corrected for sex, age, diagnostic group and number of APOE-ε4 alleles. Data are reported as mean ± standard deviation, unless indicated otherwise. Adjusted estimates of the mean and the respective standard error are reported in square brackets. Sex, age, number of APOE-ε4 alleles and clinical group were entered as nuisance covariates. For groups where variables are non-normally distributed, median (interquartile range) is also reported, in italics.

*MMSE* Mini-Mental State Examination, *SUVr* standardized uptake value ratio.

^1^Significant differences after post-hoc comparisons, at *p* < 0.05, Bonferroni-corrected for multiple comparisons:

^a^csf−/pet− vs. CSF+/pet−; csf−/pet− vs. CSF+/PET+; csf−/PET+ vs. CSF+/pet−; csf−/PET+ vs. CSF+/PET+.

^b^csf−/pet− vs. CSF+/PET+.

^c^csf−/pet− vs. CSF+/pet−; csf−/pet− vs. CSF+/PET+; csf−/PET+ vs. CSF+/pet−; csf−/PET+ vs. CSF+/PET+; CSF+/pet− vs. CSF+/PET+.

^d^csf−/pet− vs. csf−/PET+; csf−/pet− vs. CSF+/PET+; csf−/PET+ vs. CSF+/pet−; csf−/PET+ vs. CSF+/PET+; CSF+/pet− vs. CSF+/PET+.

**Fig. 2:**
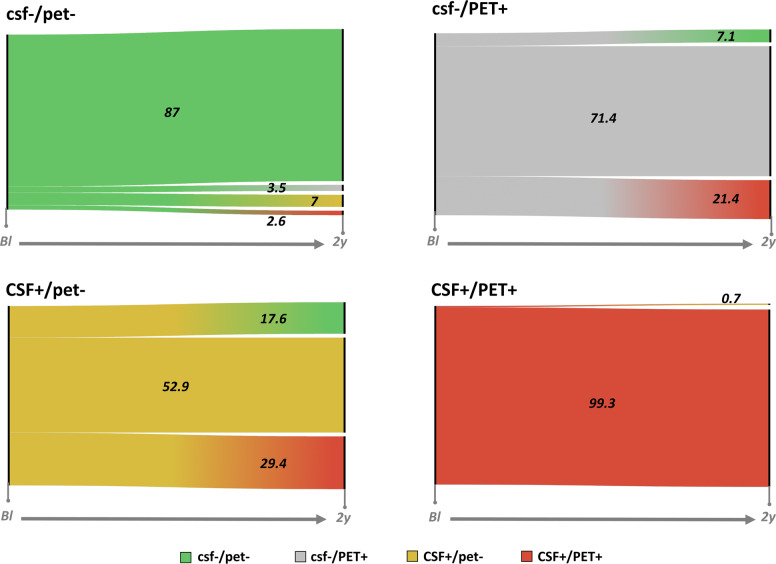
Trajectories of biomarker changes across cases with concordant and discordant amyloid-β biomarkers. Alluvial plots show trajectories of biomarker changes in csf−/pet−, csf−/PET+, CSF+/pet− and CSF+/PET+ cases (from top left corner, clockwise). Two time points are displayed for each group, i.e., baseline and 2-year follow-up. bl baseline, 2y 2-year follow-up.

The assessment of simultaneous 2-year longitudinal changes in both CSF amyloid-β_42_ and [18F]Florbetapir PET biomarkers in participants with both amyloid-β CSF and PET measurements available, obtained within 90 days, both at baseline and at 2-year follow-up (*n* = 289; Fig. 2), showed that participants with normal CSF amyloid-β_42_ and [18F]Florbetapir PET biomarkers (csf−/pet− group) were more likely to progress to isolated CSF amyloid-β_42_ positivity or to isolated [18F]Florbetapir PET positivity than to progress directly to full biomarker positivity (10.5% [*n* = 12/115] vs. 2.6% [3/115] of baseline csf−/pet− cases, *χ*^2^_(1)_ = 5.40; *p* < 0.05). The frequency of progression to full biomarker positivity was significantly higher in both CSF+/pet− and csf−/PET+ groups (25.8% [*n* = 8/31]), compared to the csf−/pet− group (2.6% [*n* = 3/115]) (*χ*^2^_(1)_ = 18.69; *p* < 0.001; *χ*^2^_(1)_ = 9.97; *p* < 0.005, respectively), with no significant differences between CSF+/pet− and csf−/PET+ groups (*χ*^2^_(1)_ = 0.26; *p* = 0.613). Finally, CSF+/PET+ participants were highly stable, with full biomarker positivity representing the endpoint of amyloid-β biomarkers changes for 99.3% [*n* = 142/143] of all cases.

At baseline (*n* = 867), when compared to the remaining groups, the groups with discordant amyloid-β biomarkers presented with “intermediate” levels of the pathological amyloid-β biomarker, i.e., (i) the CSF+/pet− group had significantly lower CSF amyloid-β_42_ levels than those with normal CSF amyloid-β_42_ values (both csf−/pet− and csf−/PET+), but significantly higher CSF amyloid-β_42_ levels as compared to the CSF+/PET+ group; (ii) the csf−/PET+ group had significantly higher [18F]Florbetapir PET retention compared to either the csf−/pet− or CSF+/pet− groups but significantly lower as compared to the CSF+/PET+ level (Table 2). This pattern was consistently observed at the regional level as well (Supplementary Fig. 1; Supplementary Table 2). We also observed a dissociation between baseline [18F]Florbetapir PET *retention* and the rate of [18F]Florbetapir PET *accumulation*, where rates of [18F]Florbetapir PET accumulation are expressed in ΔSUVr/year, as computed over 2-year (*n* = 608) and 4-year (*n* = 348) follow-up time intervals. Significant [18F]Florbetapir PET accumulation rates were reported in CSF+/PET+ [95% CI at 2 y: 0.009–0.012; 4 y: 0.011–0.013] and CSF+/pet− cases [95% CI at 2 y: 0.003–0.009; 4 y: 0.004–0.009], while minimal to null accumulation was present in csf−/pet− [95% CI at 2 y: 0.001–0.002; 4 y: 0–0.002] and csf−/PET+ [95% CI at 2 y: 0–0.003; 4 y: 0–0.005] cases (Supplementary Fig. 1; Supplementary Table 2).

When systematically comparing discordant and concordant groups considering (i) biomarkers for tau pathology and neurodegeneration, (ii) clinical and neuropsychological measures, (iii) genetic assessments and (iv) confounding factors (data available in up to *n* = 867 participants; see “Participants and Methods” for the precise values), we found that:

(i) A systematic comparison of biomarkers for tau pathology and neurodegeneration among CSF/PET groups revealed no significant differences between the CSF+/pet− and csf−/PET+ groups. Only the CSF+/PET+ group presented with increased baseline tau pathology (as measured by CSF phosphorylated-tau_181_) and neurodegeneration (as measured by CSF total-tau, [18F]Fluorodeoxyglucose PET, and MRI), and faster rate of neurodegeneration as compared to the csf−/pet− group (Supplementary Table 3).

(ii) Groups with discordant biomarkers were not at increased risk of clinical progression (csf−/PET+: hazard risk  = 1.14 [95% CI: 0.44–2.99], *p* = 0.789; CSF+/pet−: hazard risk = 1.59 [95% CI: 0.74–3.42], *p* = 0.235) as compared to the csf−/pet− group, and in contrast with the CSF+/PET+ group (hazard risk = 4.12 [95% CI: 2.59–6.56], *p* < 0.001). Discordant groups presented no difference in either baseline levels or rate of cognitive decline as measured by MMSE, ADAS-cog11 and RAVLT-learning subscale, when compared to the csf−/pet− group. Only the CSF+/PET+ group presented with significant rates of cognitive decline compared to all other groups (Supplementary Table 4). When examining the severity of neuropsychiatric depressive symptoms, we found that the GDS score was significantly higher in the csf−/PET+ group (2.09 [2.07]), as compared to all remaining groups (csf−/pet−: 1.48 [1.66]; CSF+/pet−: 1.50 [1.56]; CSF+/PET+: 1.44 [1.36]) (*F*_(3,857)_ = 2.75, *p* < 0.05) (Supplementary Table 4).

(iii) Genetic assessment revealed a significant association between number of *APOE*-ε4 alleles and CSF/PET amyloid-β biomarkers profile (*χ*^2^_(6)_ = 197; *p* < 0.001; Table 2). Although most of the association was driven by fully negative vs. fully positive cases, csf−/PET+ cases presented with a lower prevalence of *APOE*-ε4ε4 genotype than expected (0%; standardized residual = −2). Isolated PET positivity was associated with female sex (standardized residual = 2.1; *χ*^2^_(3)_ = 13.83; *p* < 0.005; Table 2).

(iv) When examining a series of confounding factors in amyloid-β fluid and molecular imaging assessments (Supplementary Table 5), we found no differences across concordant and discordant CSF/PET groups, namely: (a) size of ventricles, as a proxy for disrupted CSF clearance mechanisms [52] was not disproportionally affected in cases with discordant amyloid-β biomarkers compared to CSF+/PET+ cases (csf−/PET+: 0.02 ± 0.02; CSF+/pet−: 0.03 ± 0.02; CSF+/PET+: 0.03 ± 0.01); (b) no significant difference was reported for frequency/severity of sleep disturbances, previously associated with CSF amyloid-β_42_ changes [53] (*F*_(3,852)_ = 0.26, *p* = 0.854); (c) time interval between [18F]Florbetapir injection and start of image acquisition was not increased in cases with discordant amyloid-β biomarkers (*F*_(3,857)_ = 1.40, *p* = 0.242); (d) occurrence of infarcts (*χ*^2^_(3)_ = 2.69, *p* = 0.442) as well as volume of white matter hyperintensities (*F*_(3,808)_ = 0.57, *p* = 0.634) within the SUVr reference region did not differ between groups. Finally, lack of significantly decreased CSF amyloid-β_40_ levels in csf−/PET+ individuals (csf−/pet−: 8718.85 pg/ml; csf−/PET+: 9788.26 pg/ml), together with presence of relevant, but not severe, levels of occipital amyloid-β retention (csf−/pet−: 0.75 [0.06]; csf−/PET+: 0.83 [0.06]; CSF+/PET+: 0.94 [0.13]) makes it unlikely that csf−/PET+ cases are due to cerebral amyloid angiopathy.

## Discussion

Discordance in CSF and PET measures of amyloidosis is fairly common, occurring in ≈20% of individuals at preclinical and prodromal stages of Alzheimer’s disease [15]. Yet, guidelines on how to correctly interpret discordant CSF/PET results are lacking. In this study, we validated the hypothesis that discordance in CSF/PET amyloid-β biomarkers provides unique information and that the existence of discordant groups may result from differences in amyloid-β processing and kinetics in the CSF vs. in the brain. Through systematic assessment of discordant amyloid-β biomarker results at both cross-sectional and longitudinal levels, we showed that biomarker discordance represents a natural phase in the progression of amyloid-β pathology, with either CSF or PET amyloid-β biomarkers becoming abnormal first and not concurrently. We subsequently identified two mutually exclusive trajectories (“CSF-first” vs. “PET-first”) toward established amyloid-β pathology, characterized by different genetic profiles and rates of amyloid-β accumulation (“fast” vs. “slow” accumulators, respectively). In addition, we showed that biomarker discordance was not due to systematic errors in CSF and PET measurements. Instead, biomarker discordance carried prognostic relevance as a marker of progression toward established amyloid-β pathology, thus allowing identification of individuals at subsequent risk of clinical progression, cognitive decline, increased neurodegeneration, and tau pathology.

First, for the longitudinal cohort as a whole, decreased CSF amyloid-β_42_ levels at baseline were predictive of subsequent increases in amyloid-β plaques in the brain as measured by longitudinal [18F]Florbetapir PET (Fig. 1). This observation supports the concept that decreased CSF amyloid-β_42_ might be a proxy for the rate at which soluble amyloid-β isoforms are being trapped into non-soluble amyloid-β aggregates in the brain as measured by PET. Consistent with this observation, and in participants with normal CSF/PET amyloid-β biomarkers, CSF amyloid-β_42_, and [18F]Florbetapir PET levels did not become pathological at the same time. Preferentially, CSF amyloid-β_42_ became abnormal first without detectable brain [18F]Florbetapir PET uptake (7% of csf−/pet− cases progressed to CSF+/pet−). This evidence, coupled with the finding that about 30% of CSF+/pet− cases progressed to CSF+/PET+, qualifies isolated CSF positivity as a preferential pathway from fully normal to fully pathological amyloid-β biomarkers, consistent with previous reports [19, 24].

**Fig. 3:**
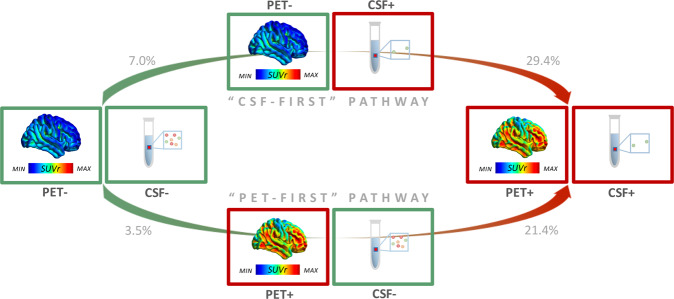
Trajectories toward full amyloid-β biomarkers abnormality. The figure shows the different trajectories toward full abnormality in amyloid-β biomarkers. Different possible pathways, i.e., either CSF amyloid-β_42_ positivity first or [18F]Florbetapir PET positivity first, are possible.

However, our cross-sectional and longitudinal analyses in this large cohort provided evidence for the existence of an additional alternative pathway, where [18F]Florbetapir PET became abnormal first. Firstly, and consistent with previous reports [6–8, 13–15, 17, 18, 20–32, 34] (Supplementary Table 1), we observed isolated [18F]Florbetapir PET positivity in a non-negligible percentage of cases. This cross-sectional evidence alone questions whether isolated CSF amyloid-β_42_ positivity represents the only possible pathway toward fully abnormal amyloid-β biomarkers. Indeed, our longitudinal results showed that—in a proportion of participants with normal amyloid-β biomarkers at baseline—[18F]Florbetapir PET became abnormal first, while levels of CSF amyloid-β_42_ were still normal (3.5% of csf−/pet− cases progressed to csf−/PET+). In addition, about 20% of the participants with isolated pathological [18F]Florbetapir PET progressed to CSF+/PET+. These longitudinal data suggest that cases with isolated [18F]Florbetapir PET positivity are part of an alternative (PET-first) pathway toward established amyloid-β pathology (Fig. 3).

Of note, the frequency of progression from csf−/pet− to either CSF+/pet− or PET+/csf− was not significantly different; this finding supports the concept that both CSF-first and PET-first pathways are relevant. Furthermore, our study found a slower rate of [18F]Florbetapir PET accumulation in the csf−/PET+ compared to the CSF+/pet− group. Thus, the existence of cases with discordant biomarkers could be partly explained by differences in amyloid-β accumulation dynamics, with csf−/PET+ and CSF+/pet− cases representing “slow” and “fast” amyloid-β accumulators, respectively. This explanation is also supported by the lower prevalence of *APOE*-ε4ε4 genotype among csf−/PET+ individuals found in this study (Table 2), considering that a slower rate of amyloid-β plaque accumulation is found in non-*APOE*-ε4 carriers compared to *APOE*-ε4 carriers [54]. In contrast, CSF+/pet− cases, despite presenting no evidence of significant amyloid-β PET tracer retention, accumulated plaques in the brain at a relatively fast rate, a finding consistent with the higher prevalence of *APOE*-ε4ε4 genotype found in the CSF+/pet− group compared to that in the csf−/PET+ group (Table 2).

On a technical note, it has been argued that occurrence of discordant findings might be inherently dependent on the choice of cutoffs to establish biomarkers’ abnormality [36]. Still, independently of the type of cutoffs adopted, previous studies consistently reported a proportion of discordant amyloid-β biomarker results, even when using cutoffs specifically designed to optimize agreement between biomarkers [14, 16–18, 20, 21, 23, 25–27, 29–31] (Supplementary Table 1). Similarly, it has been proposed that discordant cases might be due to false negative or false positive results, CSF analytical factors or failed PET scans [19]. Our assessment of a comprehensive series of confounding factors in this study argues against this explanation. Altogether, our results argue against the claim that discordant findings in amyloid-β biomarkers systematically represent false positive/negative results, suggesting instead that amyloid-β biomarkers discordance, per se, carries relevant information with respect to brain amyloid-β burden *and* accumulation rate. Most importantly, amyloid-β biomarkers discordance represents a strong predictor of future progression toward full amyloid-β biomarkers abnormalities (Fig. 2), thus allowing prompt identification of individuals that will be at higher subsequent risk for clinical progression and cognitive decline, steeper rates of neurodegeneration, and tau pathology [14, 15, 19, 28].

This study has some limitations. We acknowledge that the sample size of discordant biomarker groups was relatively limited (*n* = 62 for CSF+/pet− and *n* = 44 for csf−/PET+ cases). Although we used all longitudinal CSF/PET data available from ADNI at the time of our study, joint CSF/PET examinations were available at two longitudinal time points only, thus future studies incorporating additional longitudinal CSF/PET time points will be valuable to confirm our findings.

In conclusion, biomarker discordance allows for identification of individuals with elevated risk of progression toward fully abnormal amyloid-β biomarkers, with subsequent risk of neurodegeneration and cognitive decline. Our results also suggest that there are two alternative pathways (“CSF-first” vs. “PET-first”) toward established amyloid-β pathology, characterized by different genetic profiles and rates of amyloid-β accumulation. In conclusion, CSF and PET amyloid-β biomarkers provide complementary rather than redundant information, with potential implications for their use as biomarkers in clinical trials.

## Supplementary information

Supplementary Figure 1

Supplementary Table 1

Supplementary Table 2

Supplementary Table 3

Supplementary Table 4

Supplementary Table 5
