# Supplementary material for: Longitudinal pathways of cerebrospinal fluid and positron emission tomography biomarkers of amyloid-β positivity
Source: Mol Psychiatry. 2020 Dec 11;26(10):5864–74. doi: 10.1038/s41380-020-00950-w (PMC8758501; doi:10.1038/s41380-020-00950-w)
Supplement: Supplementary file 1 — Supplementary Figure 1 [file 41380_2020_950_MOESM1_ESM.docx]

**Supplementary Figure 1. Cortical amyloid-β burden and amyloid-β accumulation across cases with concordant and discordant amyloid-β biomarkers.**

**
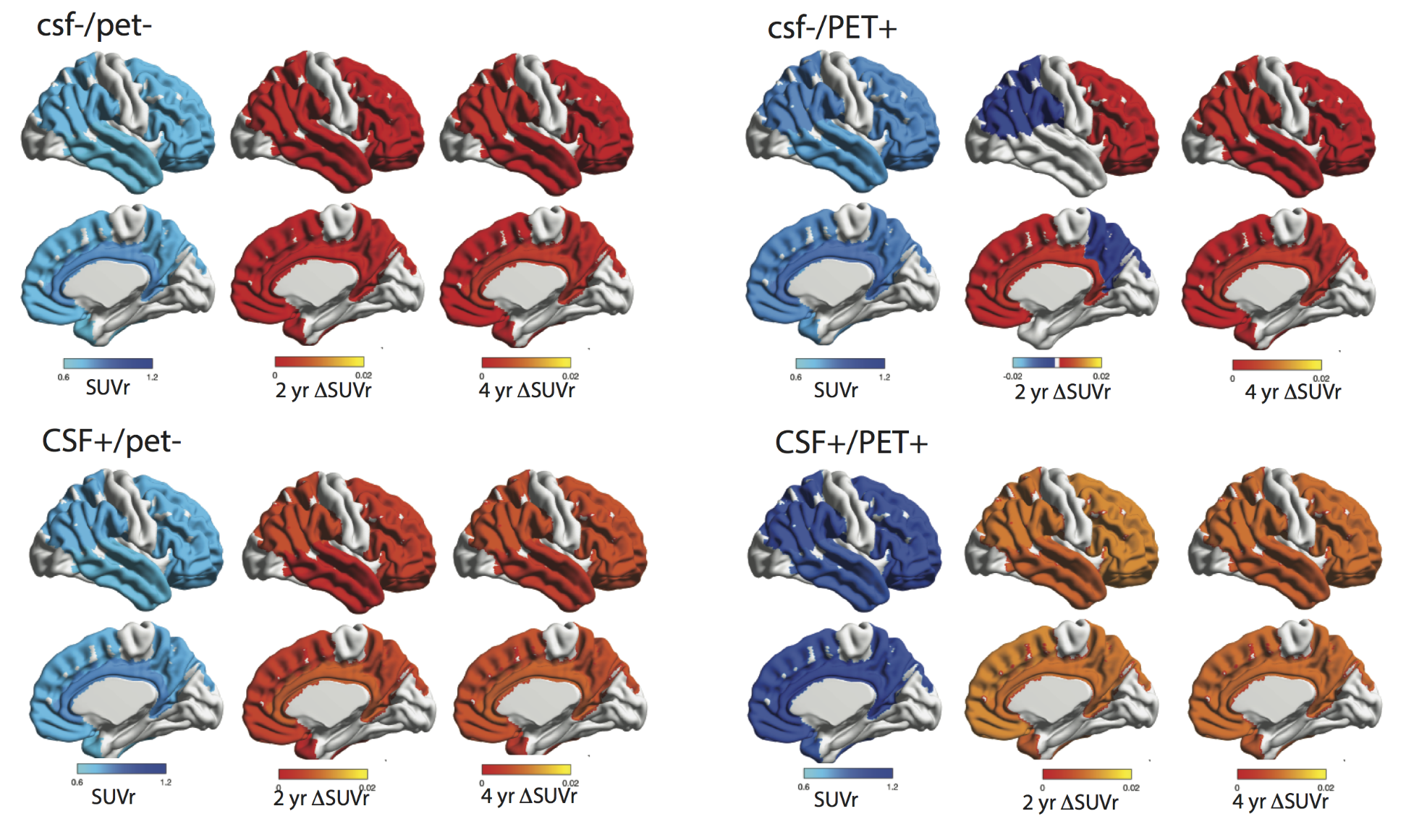
**

Brain renderings display amount of amyloid-β burden, as measured by [18F]Florbetapir PET SUVr, and rate of change in amyloid burden (ΔSUVr) over a 2-year and 4-year follow-up in csf-/pet-, csf-/PET+, CSF+/pet- and CSF+/PET+ groups (from top left corner, clockwise). Brain renderings were created using BrainNet toolbox. Abbreviations: bl = baseline; 2y = 2-year; 4y= 4-year.
