# Supplementary material for: Longitudinal pathways of cerebrospinal fluid and positron emission tomography biomarkers of amyloid-β positivity
Source: Mol Psychiatry. 2020 Dec 11;26(10):5864–74. doi: 10.1038/s41380-020-00950-w (PMC8758501; doi:10.1038/s41380-020-00950-w)
Supplement: Supplementary file 2 — Supplementary Table 1 [file 41380_2020_950_MOESM2_ESM.docx]

**Supplementary Table 1. Summary of studies reporting discordant results for CSF amyloid-β_42_ and amyloid-β PET biomarkers**

| **Study** | **Amyloid-β PET Details** | **CSF amyloid-β_42_ Details** | **Amyloid-β PET cut-off** | **CSF amyloid-β_42_ cut-off** | **PET-CSF time interval** | **Sample** | **Findings on concordant/discordant biomarkers:** |
| --- | --- | --- | --- | --- | --- | --- | --- |
| PET Amyloid Ligand [11C]PIB Uptake and Cerebrospinal Fluid -Amyloid in Mild Cognitive Impairment. Kouvinen et al, 2008 | Tracer: [11C]PiB  Quantification: SUVr  [RR: cerebellar cortex]  Global cortical uptake based on: lateral frontal cortex, lateral temporal cortex, occipital cortex, parietal cortex and posterior cingulate cortex | Quantification: immunoassay [ELISA (Innogenetics, Ghent, Belgium)] | Method: data-driven  Value: mean+2SD [value not specified] | Method: based on HC  Value: 450pg/mL | < 12 months (PET following CSF) | N=37  [N=22 HC;  N=15 MCI] | Discordant biomarkers in 8/15 (53.3%) cases:  N=1 CSF+/pet-  N=7 csf-/PET+ |
| Relationships between biomarkers in aging and dementia. Jagust et al, 2009 | Tracer: [11C]PiB  Quantification: SUVr  [RR: cerebellum]  Global cortical uptake based on: anterior cingulate, prefrontal, lateral temporal, and parietal cortex, and posterior cingulate/precuneus. | Quantification: immunoassay [multiplex xMAP Luminex plat- form (Luminex Corp, Austin TX) with Innogenetics (INNO- BIA AlzBio3, Ghent, Belgium)] | Method: independently derived [ROC approach on an independent cohort of 20 HC and 20 ADD]  Value: 1.465 SUVr | Method: independently derived [ROC approach on an independent cohort of 52 HC and 56 autopsy-confirmed ADD]  Value: 192 pg/mL | NA | N=55  [N=11 HC;  N=34 MCI  N=10 ADD] | Discordant biomarkers in 5/55 (9.09%) cases:  N=2 CSF+/pet-  N=3 csf-/PET+ |
| **Study** | **Amyloid-β PET Details** | **CSF amyloid-β_42_ Details** | **Amyloid-β PET cut-off** | **CSF amyloid-β_42_ cut-off** | **PET-CSF time interval** | **Sample** | **Findings on concordant/discordant biomarkers:** |
| Cerebrospinal fluid tau and ptau181 increase with cortical amyloid deposition in cognitively normal individuals: Implications for future clinical trials of Alzheimer's disease. Fagan et al, 2009 | Tracer: [11C]PiB  Quantification: Logan graphical analysis [RR: cerebellum]  Global cortical uptake based on: prefrontal cortex, precuneus, lateral temporal cortex and gyrus rectus. | Quantification: immunoassay [ELISA (Innogenetics, Ghent, Belgium)] | Method: NA  Value: 0.18 MCBP | Method: NA  Value: 500 pg/mL | < 24 months (PET following CSF) | N=189  [N=189 HC] | Discordant biomarkers in 28/189 (14.81%) cases:  N=28 CSF+/pet-  N=0 csf-/PET+ |
| Absence of Pittsburgh compound B detection of cerebral amyloid beta in a patient with clinical, cognitive, and cerebrospinal fluid markers of Alzheimer disease: a case report. Cairns et al, 2009 | Tracer: [11C]PiB  Quantification: Logan graphical analysis [RR: cerebellum]  Global cortical uptake based on: prefrontal cortex, lateral temporal cortex, precuneus,  occipital lobe, head of the caudate, gyrus rectus | Quantification: immunoassay [ELISA (Innogenetics, Ghent, Belgium)] | Method: NA  Value: 0.2 MCBP | Method: NA  Value: 500 pg/mL | NA | N=1  [N=1 mild ADD] | Subject was csf-/pet- at baseline 88.5 years of age) but became CSF+/pet- at follow-up (89.5 years of age)  N=1 CSF+/pet-  N=0 csf-/PET+ |
| Pittsburgh compound-B and Alzheimer's disease biomarkers in CSF, plasma and urine: An exploratory study. Gunnarson et al, 2010 | Tracer: [11C]PiB  Quantification: SUVr [RR: cerebellar cortex]  Global cortical uptake based on: frontal, parietal, temporal and posterior cingulum | Quantification: immunoassay [ELISA (Innogenetics, Ghent, Belgium)] | Method: data-driven [mean+1SD in an independent cohort of HC]  Value: 1.6 SUVr | Method: NA  Value: 450 pg/mL | NA | N=10  [N=10 mild to moderate AD] | No discordant cases  N=0 CSF+/pet-  N=0 csf-/PET+ |
| **Study** | **Amyloid-β PET Details** | **CSF amyloid-β_42_ Details** | **Amyloid-β PET cut-off** | **CSF amyloid-β_42_ cut-off** | **PET-CSF time interval** | **Sample** | **Findings on concordant/discordant biomarkers:** |
| CSF biomarker and PIB-PET-derived beta-amyloid signature predicts metabolic, gray matter, and cognitive changes in nondemented subjects. Ewers et al, 2012 | Tracer: [11C]PiB  Quantification: SUVr [RR: cerebellum]  Global cortical uptake based on: prefrontal, lateral temporal, anterior cingulate gyrus, parietal and posterior cingulate/precuneus | Quantification: immunoassay [multiplex xMAP Luminex plat- form (Luminex Corp, Austin TX) with Innogenetics (INNO- BIA AlzBio3, Ghent, Belgium)] | Method: data-driven (minimum density value in PiB bimodal distribution)  Value: 1.6 SUVr | Method: NA  Value: NA | NA | N=465  [N=124 HC;  N=229 amnestic MCI;  N=112 ADD] | Discordant biomarkers in 17/465 (3.6%) cases:  N=NA CSF+/pet-  N=NA csf-/PET+ |
| Low PiB PET retention in presence of pathologic CSF biomarkers in Arctic APP mutation carriers. Scholl et al, 2012 | Tracer: [11C]PiB  Quantification: SUVr [RR: pons]  Regional assessment in: frontal, parietal, temporal and occipital cortices, posterior cingulate, striatum, thalamus, hippocampus and cerebellum | Quantification: immunoassay [INNO-BIA AlzBio3 assay  or ELISA Innotest  (Innogenetics, Ghent, Belgium)] | Method: NA  Value: NA  *[Quantitative comparison]* | Method: data-driven [Z-score computed on HC]  Value: Z-score  < -1.645 | NA | N=23  [N=7 HC;  N=2 APParc carriers;  N=5 APParc noncarrier siblings;  N=1 PSEN1 carrier;  N=1 APPswe carrier;  N=7 sporadic ADD] | Discordant biomarkers in both APParc carriers:  N=2 CSF+/pet-  N=0 csf-/PET+ |
| **Study** | **Amyloid-β PET Details** | **CSF amyloid-β_42_ Details** | **Amyloid-β PET cut-off** | **CSF amyloid-β_42_ cut-off** | **PET-CSF time interval** | **Sample** | **Findings on concordant/discordant biomarkers:** |
| Comparing positron emission tomography imaging and cerebrospinal fluid measurements of β-amyloid. Landau et al, 2013 | Tracer: [18F]Florbetapir  Quantification: SUVr [RR: cerebellum]  Global cortical uptake based on: frontal, anterior/posterior cingulate, lateral parietal and lateral temporal regions | Quantification: immunoassay [multiplex xMAP Luminex plat- form (Luminex Corp, Austin TX) with Innogenetics (INNO- BIA AlzBio3, Ghent, Belgium)] | Method: independently derived [95% confidence interval in an independent cohort of young HC; validated for discrimination between autopsy-confirmed HC and ADD]  Value: 1.11 SUVr | Method: independently derived [derived to optimize discrimination between autopsy-confirmed HC and ADD]  Value: 192 pg/mL | < 2 weeks | N=374  [N=103 HC;  N=187 EMCI;  N=62 LMCI;  N=22 AD] | Discordant biomarkers in 52/374 (14%) cases:  N=21 CSF+/pet-  N=31 csf-/PET+ |
| Concordance between cerebrospinal fluid biomarkers and [11C]PIB PET in a memory clinic cohort. Zwan et al, 2014 | Tracer: [11C]PiB  Quantification: NA  Global cortical uptake based on: NA | Quantification: immunoassay [ELISA (Innogenetics, Ghent, Belgium)] | Method: NA  Value: NA [Visual Rating] | Method:  standard cut-off: independently derived [ROC approach on an independent cohort of 131 HC and 248 ADD]  lenient cut-off: NA  Value:  standard cut-off:550 pg/mL  lenient cut-off:640 pg/mL | 4±3 months (mean±SD) | N=136  [N=16 HC;  N=22 MCI;  N=64 ADD;  N=34 non-ADD] | Standard cut-off:  Discordant biomarkers in 22/136 (16%) cases: mostly csf-/PET+ cases  Lenient cut-off:  Discordant biomarkers in 14/136 (10%) cases: mostly CSF+/pet- cases |
| **Study** | **Amyloid-β PET Details** | **CSF amyloid-β_42_ Details** | **Amyloid-β PET cut-off** | **CSF amyloid-β_42_ cut-off** | **PET-CSF time interval** | **Sample** | **Findings on concordant/discordant biomarkers:** |
| Using florbetapir positron emission tomography to explore cerebrospinal fluid cut points and gray zones in small sample sizes. Weston et al, 2015 | Tracer: [18F]Florbetapir  Quantification: SUVr [RR: whole cerebellum]  Global cortical uptake based on: frontal, temporal, parietal, anterior cingulate, posterior cingulate, and precuneus | Quantification: immunoassay [INNOTEST (Fujirebio, Ghent, Belgium)] | Method: independently derived [derived as the 95% confidence interval in an independent cohort of young healthy controls]  Value: 1.10 SUVr + [Visual rating] | Method: data-driven [*ROC approach to optimize agreement between CSF and PET]*  Value: 630 ng/L | NA | N=23  [N=4 HC;  N=19 various dementia syndromes ] | Among cases with concordant Aβ-PET readings (N=20), discordant biomarkers were reported in 1/20 (5%) cases:  N=1 csf-/PET+  N=0 CSF+/pet- |
| Diagnostic value of cerebrospinal fluid Aβ ratios in preclinical Alzheimer’s disease. Adamczuk et al, 2015 | Tracer: [18F]Flutemetamol  Quantification: SUVr [RR: cerebellar gray matter]  Global cortical uptake based on: frontal, parietal, lateral temporal, and anterior and posterior cingulate cortices | Quantification: immunoassay [INNOTEST (Fujirebio, Ghent, Belgium)] | Method: independently derived [derived from a neuropathological study on an independent cohort of 68 subjects; positivity established if mean Bielschowsky score was > 1.5 in at least one region]  Value: 1.57 SUVr | Method: data-driven [*ROC approach to optimize agreement between CSF and PET]*  Value: 745 | NA | N=38  [N=38 HC] | Discordant biomarkers in 8/38 (21%) cases:  N=8 CSF+/pet-  N=0 csf-/PET+ |
| **Study** | **Amyloid-β PET Details** | **CSF amyloid-β_42_ Details** | **Amyloid-β PET cut-off** | **CSF amyloid-β_42_ cut-off** | **PET-CSF time interval** | **Sample** | **Findings on concordant/discordant biomarkers:** |
| Independent information from cerebrospinal fluid amyloid-β and florbetapir imaging in Alzheimer’s disease. Mattsson et al, 2015 | Tracer: [18F]Florbetapir  Quantification: SUVr [RR: whole cerebellum]  Global cortical uptake based on: lateral and medial frontal, anterior and posterior cingulate, lateral parietal, and lateral temporal regions | Quantification: immunoassay [multiplex xMAP Luminex plat- form (Luminex Corp, Austin TX) with Innogenetics (INNO- BIA AlzBio3, Ghent, Belgium)] | Method: independently derived [derived as the 95% confidence interval in an independent cohort of young healthy controls; validated for discrimination between autopsy-confirmed HC and ADD]  Value: 1.11 SUVr | Method: independently derived [ROC approach on an independent cohort of 52 HC and 56 autopsy-confirmed ADD]  Value: 192 pg/mL | NA [all measures acquired at “baseline”] | N=769  [N=161 HC;  N=68 SCC;  N=269 EMCI;  N=150 LMCI;  N=121 ADD] | Discordant biomarkers in 107/769 (13.91%) cases, mostly CSF+/pet-:  N=NA CSF+/pet-  N=NA csf-/PET+ |
| Accuracy of Brain Amyloid Detection in Clinical Practice Using Cerebrospinal Fluid β-Amyloid 42 A Cross-Validation Study Against Amyloid Positron Emission Tomography. Palmqvist et al, 2015 | Tracer: [18F]Flutemetamol  Quantification: SUVr [RR: cerebellar cortex]  Global cortical uptake based on: prefrontal, parietal, temporal lateral, anterior cingulate, posterior cingulate, and precuneus | Quantification: immunoassays [INNOTEST, Innogenetics] | Method: data-driven [derived using Gaussian Mixture Modelling]  Value: 1.42 SUVr | Method:  data-driven [*ROC approach to optimize agreement between CSF and PET*]  Value:  647 pg/mL | NA (some patients had CSF-PET assessments at >6 months) | Original cohort N=118  [N=63 SCC;  N=55 MCI]  Validation cohort  N=38  [N=17 SCC;  N=21 MCI] | Original cohort: Discordant biomarkers in 9/118 (7.62%) cases:  N=6 CSF+/pet-  N=3 csf-/PET+  Validation cohort:  Discordant biomarkers in 2/38 (5.26%) cases:  N=1 CSF+/pet-  N=1 csf-/PET+ |
| **Study** | **Amyloid-β PET Details** | **CSF amyloid-β_42_ Details** | **Amyloid-β PET cut-off** | **CSF amyloid-β_42_ cut-off** | **PET-CSF time interval** | **Sample** | **Findings on concordant/discordant biomarkers:** |
| Use of amyloid-PET to determine cutpoints for CSF markers A multicenter study. Zwan et al, 2015 | Tracer: [11C]PiB  Quantification: NA  Global cortical uptake based on: NA | Quantification: immunoassay: ELISA Innotest  (Innogenetics, Ghent, Belgium)] | Method: NA  Value: [Visual Rating] | Method:  Clinical-based cut-off: independently derived  Value: 400-550 pg/mL (depending on the centre)  PET-based cut-off:  data-driven [*ROC approach to optimize agreement between CSF and PET*]  Value: 521-616 pg/mL (depending on the centre) | 74 ± 76 days [mean±SD]  (< 1 year) | N=433  [N=57 HC;  N=99 MCI;  N=195 ADD;  N=82 non-ADD] | Clinical-based cut-off:  Discordant biomarkers in 104/433 (24%) cases:  N=NA CSF+/pet-  N=NA csf-/PET+  PET-based cut-off:  Discordant biomarkers in 68/433 (16%) cases:  N=33 CSF+/pet-  N=35 csf-/PET+ |
| **Study** | **Amyloid-β PET Details** | **CSF amyloid-β_42_ Details** | **Amyloid-β PET cut-off** | **CSF amyloid-β_42_ cut-off** | **PET-CSF time interval** | **Sample** | **Findings on concordant/discordant biomarkers:** |
| CSF Aβ42/Aβ40 and Aβ42/Aβ38 ratios: Better diagnostic markers of Alzheimer disease. Janelidze et al, 2016 | Tracer: [18F]Flutemetamol  Quantification: SUVr [RR: cerebellar cortex]  Global cortical uptake based on: prefrontal, parietal, lateral temporal, medial temporal, sensorimotor, occipital, anterior cingulate, posterior cingulate/precuneus | Quantification: immunoassays [Euroimmun (EUROIMMUN AG, Leubeck, Germany)), Meso Scale Discovery (Rockville, MD), Quanterix (Quanterix, Lexington, MA)] | Method: data-driven [derived using Gaussian Mixture Modelling from a subsample of patients]  Value: 1.42 SUVr | Method:  data-driven [*ROC approach to optimize agreement between CSF and PET*]  Value:  495.9-1742 pg/mL (depending on the immunoassay for CSF quantification) | NA (all measures acquired at “baseline”) | N=215  [N=215 MCI] | Euroimmun Immunoassay:  Discordant biomarkers in 36/215 (16.74%) cases:  N=18 CSF+/pet-  N=18 csf-/PET+  Meso Scale Discovery Immunoassay:  Discordant biomarkers in 28/215 (13.02%) cases:  N=16 CSF+/pet-  N=12 csf-/PET+  Quanterix immunoassay:  Discordant biomarkers in 17/70 (24.29%) cases:  N=8 CSF+/pet-  N=9 csf-/PET+ |
| Cerebrospinal fluid analysis detects cerebral amyloid-β accumulation earlier than positron emission tomography. Palmqvist et al, 2016 | Tracer: [18F]Florbetapir  Quantification: SUVr [RR: composite reference region -cerebellum, brainstem/pons, and subcortical white matter]  Global cortical uptake based on: frontal, lateral parietal, lateral temporal and cingulate regions | immunoassay [multiplex xMAP Luminex plat- form (Luminex Corp, Austin TX) with Innogenetics (INNO- BIA AlzBio3, Ghent, Belgium)] | Method: independently derived [adaptation of the 1.11 cut-off, as the 95% confidence interval in a cohort of young HC; validated for discrimination between autopsy-confirmed HC and ADD]  Value: 0.79 SUVr ±5% (borderline cases excluded) | Method: independently derived [ROC approach on an independent cohort of 52 HC and 56 autopsy-confirmed ADD]  Value: 192 pg/mL ±5% (borderline cases excluded) | 12 days on average (<129 days) | N=437 [N=353 after exclusion of borderline cases]  [N=114 HC;  N=165 EMCI;  N=95 LCMI] | Discordant biomarkers in 26/353 (7.37%) cases:  N=26 CSF+/pet-  N=0 csf-/PET+  [after exclusion of borderline cases] |
| **Study** | **Amyloid-β PET Details** | **CSF amyloid-β_42_ Details** | **Amyloid-β PET cut-off** | **CSF amyloid-β_42_ cut-off** | **PET-CSF time interval** | **Sample** | **Findings on concordant/discordant biomarkers:** |
| Pittsburgh compound B imaging and cerebrospinal fluid amyloid-β in a multicentre European memory clinic study. Leuzy et al, 2016 | Tracer: [11C]PiB  Quantification: SUVr [RR: cerebellar gray matter]  Global cortical uptake based on: frontal, temporal, parietal, occipital, parahippocampal, anterior and posterior cingulate regions | Quantification: immunoassay  [ELISA (INNOTEST, Fujirebio-Europe), Belgium); MSD ELISA]  absolute quantification [MS] | Method: independently derived  [derived from the upper 95% confidence limit from an independent population of HC]  Value: 1.41 SUVr (=34 CENTILOID)  + [Visual Rating] | Method:  INNOTEST:  data-driven [*ROC approach to optimize agreement between CSF and PET, derived from a similar population (see Zwan et al, 2015)*]  MSD: data-driven [Gaussian Mixture Modelling]  MS: data-driven [Gaussian Mixture Modelling]  Value:  INNOTEST:557 pg/mL  MSD: 515 pg/mL  MS: 896 pg/mL | 2.94 months (mean) | N=243  [N=13 HC;  N=81 MCI;  N=122 ADD;  N=27 non-ADD] | PET Visual:  INNOTEST: Discordant biomarkers in 63/243 (25.92%) cases:  N=26 CSF+/pet-  N=37 csf-/PET+  MSD: Discordant biomarkers in 49/243 (20.16%) cases:  N=39 CSF+/pet-  N=10 csf-/PET+  MS: Discordant biomarkers in 56/243 (23.04%) cases:  N=46 CSF+/pet-  N=10 csf-/PET+  PET Centiloid:  INNOTEST: Discordant biomarkers in 67/243 (27.57%) cases:  N=18 CSF+/pet-  N=30 csf-/PET+  MSD: Discordant biomarkers in 52/243 (21.4%) cases:  N=42 CSF+/pet-  N=10 csf-/PET+  MS: Discordant biomarkers in 60/243 (24.69%) cases:  N=50 CSF+/pet-  N=10 csf-/PET+ |
| **Study** | **Amyloid-β PET Details** | **CSF amyloid-β_42_ Details** | **Amyloid-β PET cut-off** | **CSF amyloid-β_42_ cut-off** | **PET-CSF time interval** | **Sample** | **Findings on concordant/discordant biomarkers:** |
| Cerebrospinal Fluid Aβ42/40 Corresponds Better than Aβ42 to Amyloid PET in Alzheimer’s Disease. Lewczuk et al, 2016 | Tracer: [11C]PiB  Quantification: Logan graphical analysis [RR: cerebellum]  Global cortical uptake based on: precuneus, rostral middle frontal cortex, superior frontal cortex, superior temporal cortex, middle temporal cortex, lateral orbitofrontal cortex, and medial orbitofrontal cortex | Quantification: immunoassay [INNOTEST ELISA (Fujirebio, Ghent, Belgium) | Method: NA  Value: 0.18 MCBP | Method: data-driven [*ROC approach to optimize agreement between CSF and PET]*  Value: 735 pg/mL | 12 months (PET following CSF) | N=200  [cognitively normal and abnormal participants] | Discordant biomarkers in 50/200 (25%) cases:  N=41 CSF+/pet-  N=9 csf-/PET+ |
| The Cerebrospinal Fluid Aβ1–42/Aβ1–40 Ratio Improves Concordance with Amyloid-PET for Diagnosing Alzheimer’s Disease in a Clinical Setting. Niemantsverdriet et al, 2017 | Tracer: [18F]Florbetapir  Quantification:  Vt [two-tissue compartment model with metabolite-corrected plasma input function]  SUVr [RR: cerebellum + subcortical white matter]  Global cortical uptake based on: frontal, parietal and temporal lobes | Quantification: immunoassay [INNOTEST ELISA (Fujirebio, Ghent, Belgium | Method: data-driven [ROC curve analysis to discriminate between HC and AD]  Value:  Vt= NA  SUVrCBL=1.203  SUVr_WM_=0.485  + [Visual Rating] | Method: independently derived [in-house validated cutoff values (in autopsy-confirmed ADD vs HC]  Value: 638.5 pg/mL | NA (all measures acquired at “baseline”) | N=78  [N=13 HC;  N=48 MCI;  N=17 ADD] | Vt= Discordant biomarkers in 13/78 (16.67%) cases:  N=2 CSF+/pet-  N=11 csf-/PET+  SUVr_CBL_= Discordant biomarkers in 22/78 (28.21%) cases:  N=0 CSF+/pet-  N=22 csf-/PET+  SUVr_WM_= Discordant biomarkers in 25/78 (32.05%) cases:  N=0 CSF+/pet-  N=25 csf-/PET+  Visual Rating Discordant biomarkers in 18/78 (23.08%) cases:  N=4 CSF+/pet-  N=14 csf-/PET+ |
| **Study** | **Amyloid-β PET Details** | **CSF amyloid-β_42_ Details** | **Amyloid-β PET cut-off** | **CSF amyloid-β_42_ cut-off** | **PET-CSF time interval** | **Sample** | **Findings on concordant/discordant biomarkers:** |
| Earliest accumulation of β-amyloid occurs within the default-mode network and concurrently affects brain connectivity. Palmqvist et al, 2017 | Tracer: [18F]Florbetapir  Quantification: SUVr [RR: composite reference region -cerebellum, brainstem/pons, and subcortical white matter]  Global cortical uptake based on: NA | immunoassay [multiplex xMAP Luminex plat- form (Luminex Corp, Austin TX) with Innogenetics (INNO- BIA AlzBio3, Ghent, Belgium)] | Method: data-driven [Gaussian Mixture Modelling]  Value: 0.872 SUVr*  *for PVE-corrected images | Method: independently derived [ROC approach on an independent cohort of 52 HC and 56 autopsy-confirmed ADD]  Value: 192 pg/mL | 9.6 days on average (<110 days) | N=473  [N=176 HC;  N=297 MCI] | Discordant biomarkers in 64/768 (13.53%) cases:  N=59 CSF+/pet-  N=5 csf-/PET+ |
| Clinic-Based Validation of Cerebrospinal  Fluid Biomarkers with Florbetapir PET  for Diagnosis of Dementia. Alvarez et al, 2018 | Tracer: [18F]Florbetapir  Quantification: NA  Global cortical uptake based on: NA | Quantification: immunoassay [INNOTEST ELISA (Fujirebio, Ghent, Belgium)] | Method: NA  Value: [Visual Rating] | Method: data-driven [*ROC approach to optimize agreement between CSF and PET]*  Value:  Balanced sensitivity/specificity: 629 pg/mL  Youden Index: 962 pg/mL  80% specificity: 541 pg/mL | 48 days on average | N=68  [N=50 MCI;  N=11 ADD  N=7 non-ADD] | Balanced sensitivity/specificity: Discordant biomarkers in 14/68 (20.58%) cases:  N=1 CSF+/pet-  N=13 csf-/PET+  Youden Index: Discordant biomarkers in 4/68 (5.88%) cases:  N=3 CSF+/pet-  N=1 csf-/PET+  80% specificity: Discordant biomarkers in 16/68 (23.52%) cases:  N=1 CSF+/pet-  N=15 csf-/PET+ |
| **Study** | **Amyloid-β PET Details** | **CSF amyloid-β_42_ Details** | **Amyloid-β PET cut-off** | **CSF amyloid-β_42_ cut-off** | **PET-CSF time interval** | **Sample** | **Findings on concordant/discordant biomarkers:** |
| Concordance Between Cerebrospinal  Fluid Biomarkers with Alzheimer’s  Disease Pathology Between Three  Independent Assay Platforms. Doecke et al, 2018 | Tracer: [11C]PiB (N= 28); [18F]Flutemetamol (N = 32); [18F]Florbetapir (N= 17)  Quantification: SUVr (RR: whole cerebellum for [11C]PiB and [18F]Florbetapir images; pons for [18F]Flutemetamol  Global cortical uptake based on: frontal, superior parietal,  lateral temporal, lateral occipital, and anterior and  posterior cingulate regions | Quantificaiton: Immunoassay [INNO-BIA AlzBio3 xMAP; INNOTEST ELISA (Fujirebio, Ghent, Belgium); EUROIMMUNE-ADx (NeuroSciences Ghent, Belgium)] | Method: independently-derived [cluster analysis on an independent sample of 122 HC]  Value: 1.4 SUVR [after transformation of all SUVrs into PiB-like SUVR using Before the Centiloid Kernel Transformation] | Method: various [*including* *an Expectation Maximization approach to optimize agreement between CSF and PET]*  Value:  xMAP: 416 pg/mL  INNOTEST: 544 pg/mL  EUROIMMUNE: 649 pg/mL | NA | N=77  [N=48 HC;  N=15 MCI;  N=14 ADD] | xMAP: Discordant biomarkers in 13/77 (16.88%) cases:  N=6 CSF+/pet-  N=7 csf-/PET+  INNOTEST: Discordant biomarkers in 15/77 (19.48%) cases:  N=3 CSF+/pet-  N=12 csf-/PET+  EUROIMMUNE: Discordant biomarkers in 9/77(11.69%) cases:  N=3 CSF+/pet-  N=6 csf-/PET+ |
| **Study** | **Amyloid-β PET Details** | **CSF amyloid-β_42_ Details** | **Amyloid-β PET cut-off** | **CSF amyloid-β_42_ cut-off** | **PET-CSF time interval** | **Sample** | **Findings on concordant/discordant biomarkers:** |
| CSF biomarkers of Alzheimer’s disease concord with amyloid-β PET and  predict clinical progression: A study of fully automated immunoassays  in BioFINDER and ADNI cohorts. Hansson et al, 2018 | Tracer: [18F]Flutemetamol (N=277) [18F]Florbetapir (N=646)  Quantification: SUVr (RR: whole cerebellum)  Global cortical uptake based on: lateral temporal, frontal, posterior cingulate/precuneus, and parietal cortices | Quantification: ElectroChemiLuminescence Immunoassay (ELECSYS) | Method: data-driven [Gaussian Mixture Modelling]  Value: [18F]Futemetamol: 1.24 SUVr;  [18F]Florbetapir: 1.16 SUVr  + [Visual Rating] | Method: data-driven [*ROC approach to optimize agreement between CSF and PET]*  Value:  [18F]Flutemetamol cohort: 1100 pg/mL  [18F]Florbetapir cohort: 880 pg/mL | NA | N=923  [N=214 SCC;  N=4 unclassified (SCC/MCI):  N=577 MCI;  N=128 ADD] | Visual Rating:  [18F]Flutemetamol cohort: Discordant biomarkers in 56/277(20.2%) cases:  N=46 CSF+/pet-  N=10 csf-/PET+  [18F]Florbetapir cohort: Discordant biomarkers in 101/646(15.6%) cases:  N=44 CSF+/pet-  N=57 csf-/PET+  Quantitative assessment:  [18F]Flutemetamol cohort: Discordant biomarkers in 38/277(13.7%) cases:  N=NA CSF+/pet-; N=NA csf-/PET+  [18F]Florbetapir cohort: Discordant biomarkers in 120/646(18.6%) cases:  N=NA CSF+/pet-; N=NA csf-/PET+ |
| Amyloid-β PET—Correlation with  cerebrospinal fluid biomarkers and prediction  of Alzheimer´s disease diagnosis in a memory  clinic. Gloersen-Muller et al, 2019 | Tracer: [18F]Flutemetamol  Quantification: NA  Global cortical uptake based on:  NA | Quantification: immunoassay [INNOTEST ELISA (Fujirebio, Ghent, Belgium)] | Method: NA  Value: [Visual Rating] | Method: NA [cut-off routinely used] + *ROC approach to optimize agreement between CSF and PET]*  Value:  Standard cut-off: 550 pg/mL  Data-driven cut-off:706.5 pg/mL | <190 days | N=64  [N=5 SCD;  N=17 MCI;  N=34 ADD  N=8 non-ADD] | Standard cut-off: Discordant biomarkers in 21/64(32.81%) cases:  N=1 CSF+/pet-  N=20 csf-/PET+  Data-driven cut-off: Discordant biomarkers in 8/64 (12.5%) cases:  N=4 CSF+/pet-  N=4 csf-/PET+ |
| **Study** | **Amyloid-β PET Details** | **CSF amyloid-β_42_ Details** | **Amyloid-β PET cut-off** | **CSF amyloid-β_42_ cut-off** | **PET-CSF time interval** | **Sample** | **Findings on concordant/discordant biomarkers:** |
| Agreement of amyloid PET and CSF biomarkers for  Alzheimer’s disease on Lumipulse. Alcolea et al, 2019 | Tracer: [18F]Florbetapir  Quantification: SUVr [RR: whole cerebellum]  Global cortical uptake based on: frontal, lateral parietal, lateral temporal and anterior/  posterior cingulate. | Quantification: immunoassay [LUMIPULSE G600II automated  platform (Fujirebio, Ghent, Belgium)] | Method: independently derived [derived as the 95% confidence interval in an independent cohort of young healthy controls; validated for discrimination between autopsy-confirmed HC and ADD]  Value: 1.11 SUVr + [Visual Rating] | Method: data-driven [*ROC approach to optimize agreement between CSF and PET]*  Value: 916pg/mL | 152±86 days [mean±SD] | N=94  [N=6 HC;  N=35 MCI;  N=12 ADD  N=41 non-ADD] | Visual Rating: Discordant biomarkers in 20/94 (21%) cases:  N=17 CSF+/pet-  N=3 csf-/PET+  Quantitative evaluation: Similar results as those obtained as for visual rating |
| Testing the 2018 NIA-AA research  framework in a retrospective large cohort of patients with cognitive impairment: from  biological biomarkers to clinical syndromes. Carandini et al, 2019 | Tracer: [18F]Florbetapir [N=24]  Quantification: SUVr [RR: whole cerebellum]  Global cortical uptake based on:  anterior cingulate gyrus, frontal lobe, parietal lobe, posterior cingulate gyrus, precuneus, temporal lobe | Quantification: immunoassay ELISAs [Innotest assays, Fujirebio (formerly Innogenetics) | Method: independently derived [derived as the 95% confidence interval in an independent cohort of young healthy controls; validated for discrimination between autopsy-confirmed HC and ADD]  Value: 1.11 SUVr | Method: data-driven [*ROC approach to optimize agreement between CSF and PET]*  Value: 660 pg/mL | Approximately 6 months (<1 year) | N=44  [N= 24 MCI;  N= 14 ADD;  N= 2 CAA;  N= 4 non-ADD] | Discordant biomarkers in 6/44 (13.63%) cases:  N=2 CSF+/pet-  N=4 csf-/PET+ |
| **Study** | **Amyloid-β PET Details** | **CSF amyloid-β_42_ Details** | **Amyloid-β PET cut-off** | **CSF amyloid-β_42_ cut-off** | **PET-CSF time interval** | **Sample** | **Findings on concordant/discordant biomarkers:** |
| Assessment of 18F-Florbetaben Amyloid  PET Imaging in Patients with Suspected  Alzheimer’s Disease and Isolated Increase  in Cerebrospinal Fluid Tau Proteins. Manca et al, 2019 | Tracer: [18F]Florbetaben  Quantification: NA  Global cortical uptake based on: NA | Quantification: immunoassay ELISA [INNOTEST  (Fujirebio, Ghent, Belgium)] | Method: NA  Value: BAPL>1 [Visual Rating] | Method: NA [cut-off routinely used in clinical practice]  Value: 600pg/mL | NA | N=34  [N=18 mild neurocognitive disorder;  N=16 suspected ADD]*  **with abnormal p-tau/t-tau but normal Aβ42* | Discordant biomarkers in 8/34 (23.53%) cases:  N=0 CSF+/pet-  N=8 csf-/PET+ |
| Role of Cerebrospinal Fluid Biomarkers and (18)F-florbetapir  PET Imaging in the Diagnosis of Primary Progressive Aphasia  A Retrospective Analysis. Perini et al, 2019 | Tracer: [18F]Florbetaben  Quantification: NA  Global cortical uptake based on: NA | Quantification: immunoassay ELISA [(Fujirebio, Ghent, Belgium) ] | Method: NA  Value: NA [Visual Rating] | Method: independently derived [cut-off yielding 0.85 sensitivity in discriminating HC and ADD, derived in an independent cohort]  Value: 482pg/mL | NA | N=6  [N=6 PPA] | Discordant biomarkers in 0/6 (0%) cases:  N=0 CSF+/pet-  N=0 csf-/PET+ |
| Incremental value of amyloid-PET versus CSF in the diagnosis  of Alzheimer’s disease. Ramusino et al, 2019 | Tracer: [18F]Florbetaben  Quantification: NA  Global cortical uptake based on: NA | Quantification: immunoassay ELISA [INNOTEST, Fujirebio, Ghent, Belgium/ EUROIMMUN, Euroimmun, Lubeck, Germany] | Method: NA  Value: [Visual Rating] | Method: NA  Value: 600-650 pg/mL [depending on the clinical centre] | < 6 months | N=71  [N=60 MCI;  N=11 ADD] | Discordant biomarkers in 17/71 (23.94%) cases:  N=6 CSF+/pet-  N=11 csf-/PET+ |
| **Study** | **Amyloid-β PET Details** | **CSF amyloid-β_42_ Details** | **Amyloid-β PET cut-off** | **CSF amyloid-β_42_ cut-off** | **PET-CSF time interval** | **Sample** | **Findings on concordant/discordant biomarkers:** |
| CSF biomarkers and amyloid PET: concordance and diagnostic  accuracy in a MCI cohort. Spallazzi et al, 2019 | Tracer: [18F]Florbetaben  Quantification: NA  Global cortical uptake based on: NA | Quantification: immunoassay ELISA [Fujirebio, Ghent, Belgium] | Method: NA  Value: BAPL>1 [Visual Rating] | Method: NA  Value:  Standard cut-off:600pg/mL  Lenient cut-off[1]:550pg/mL  Lenient cut-off[2]:450pg/mL | < 6 months | N=31  [N=31 MCI] | Standard cut-off + Lenient cut-off[1]:: Discordant biomarkers in 7/31 (22.6%) cases:  N=NA CSF+/pet-  N=NA csf-/PET+  Lenient cut-off[2]: Discordant biomarkers in 8/31 (25.9%) cases:  N=NA CSF+/pet-  N=NA csf-/PET+ |
| Discordant amyloid-β PET and CSF biomarkers and its clinical consequences. Wilde & Reimand et al, 2019 | Tracer: [11C]PiB [N=271]  [18F]Florbetapir [N=24]  [18F]Florbetaben [N=322]  [18F]Flutemetamol [N= 151]  Quantification: NA  Global cortical uptake based on: NA | Quantification: immunoassay ELISAs [Innotest assays, Fujirebio (formerly Innogenetics) | Method: NA  Value: [Visual Rating] | Method: data-driven (derived from Gaussian mixture modeling in a partially overlapping population; cross-validated against Aβ-PET results)  Cut-off: 813 pg/mL | 54±75 days [mean±SD] (< 1 year) | N=768  [N=194 SCD;  N=127 MCI;  N=309 ADD;  N=138 non-ADD] | Discordant biomarkers in 97/768 (12.63%) cases:  N=65 CSF+/pet-  N=32 csf-/PET+ |

*Abbreviations: [11C] = Carbon-11; [18F] = Fluorine-18; ADD=Alzheimer´s disease dementia; APOE=apolipoprotein E; APP= amyloid precursor protein; APParc=* *Arctic APP; APPswe=Swedish APP; BAPL=brain amyloid plaque load; CBL= cerebellum; EMCI= early MCI; HC=healthy control; LMCI= late MCI; MCBP= mean cortical binding potential; MCI=mild cognitive impairment; MMSE=Mini-Mental State Examination; MS=mass spectrometry;PSEN1= presenilin-1; PVE=partial volume effect; ROC=Receiver Operating Characteristic; RR= Reference Region; SCD=subjective cognitive complaints; SUVr=standardized uptake value ratio; Vt= total distribution volume; WM=white matter*
