# Supplementary material for: Longitudinal pathways of cerebrospinal fluid and positron emission tomography biomarkers of amyloid-β positivity
Source: Mol Psychiatry. 2020 Dec 11;26(10):5864–74. doi: 10.1038/s41380-020-00950-w (PMC8758501; doi:10.1038/s41380-020-00950-w)
Supplement: Supplementary file 3 — Supplementary Table 2 [file 41380_2020_950_MOESM3_ESM.docx]

**Supplementary Table 2. Baseline amyloid-β PET SUVr and longitudinal rates of change of amyloid-β PET SUVr in concordant and discordant biomarker groups**

|  | **csf-/pet-** | **csf-/PET+** | **CSF+/pet-** | **CSF+/PET+** | **Test value;** **p** | **Post-hoc comparison** |
| --- | --- | --- | --- | --- | --- | --- |
| **Baseline amyloid-β PET SUVr** *(global)* | 0.73±0.03  [0.75±2.9E-03] | 0.82±0.03  [0.83±5.88E-03]  *0.81 (0.80 – 0.83)* | 0.75±0.03  [0.76±4.41E-03] | 1±0.1  [0.99±5E-03] | F_(3,859)_=  487.61; p<0.001 | csf-/pet- < CSF+/PET+  csf-/PET+ < CSF+/PET+  CSF+/pet- < CSF+/PET+  csf-/pet- < csf-/PET+  CSF+/pet- < csf-/PET+ |
| **N** | 300 | 44 | 62 | 461 | - | - |
| **Baseline amyloid-β PET SUVr** *(frontal)* | 0.72±0.03  [0.74±3.23E-03] | 0.81±0.05  [0.82±7.85E-03]  *0.80 (0.78 – 0.82)* | 0.74±0.03  [0.75±5.01E-03] | 1±0.11  [0.99±5.62E-03] | F_(3,859)_=  433.95; p<0.001 | csf-/pet- < CSF+/PET+  csf-/PET+ < CSF+/PET+  CSF+/pet- < CSF+/PET+  csf-/pet- < csf-/PET+  CSF+/pet- < csf-/PET+ |
| **N** | 300 | 44 | 62 | 461 | - | - |
| **Baseline amyloid-β PET SUVr** *(cingulate)* | 0.80±0.04  [0.81±3.45E-03] | 0.87±0.05  [0.88±8.33E-03] | 0.81±0.04  [0.82±5.44E-03] | 1.07±0.11  [1.05±5.23E-03] | F_(3,859)_=  435.47; p<0.001 | csf-/pet- < CSF+/PET+  csf-/PET+ < CSF+/PET+  CSF+/pet- < CSF+/PET+  csf-/pet- < csf-/PET+  CSF+/pet- < csf-/PET+ |
| **N** | 300 | 44 | 62 | 461 | - | - |
| **Baseline amyloid-β PET SUVr** *(parietal)* | 0.73±0.04  [0.75±3.65E-03] | 0.83±0.05  [0.84±8.12E-03]  *0.83 (0.80 – 0.85)* | 0.75±0.04  [0.76±5.8E-03] | 1±0.11  [1±5.59E-03] | F_(3,859)_=  425.39; p<0.001 | csf-/pet- < CSF+/PET+  csf-/PET+ < CSF+/PET+  CSF+/pet- < CSF+/PET+  csf-/pet- < csf-/PET+  CSF+/pet- < csf-/PET+ |
| **N** | 300 | 44 | 62 | 461 | - | - |
|  | **csf-/pet-** | **csf-/PET+** | **CSF+/pet-** | **CSF+/PET+** | **Test value;** **p** | **Post-hoc comparison** |
| **Baseline amyloid-β PET SUVr** *(temporal)* | 0.69±0.03  [0.70±3.15E-03] | 0.75±0.03  [0.76±6.35E-03] | 0.70±0.03  [0.71±4.96E-03] | 0.93±0.1  [0.92±5.3E-03] | F_(3,859)_=  356.5; p<0.001 | csf-/pet- < CSF+/PET+  csf-/PET+ < CSF+/PET+  CSF+/pet- < CSF+/PET+  csf-/pet- < csf-/PET+  CSF+/pet- < csf-/PET+ |
| **N** | 300 | 44 | 62 | 461 | - | - |
| **Annual rate of change of**  **amyloid-β PET SUVr over 2 yr**  *(global; ΔSUVr/yr)* | 2E-03±0.01  [(2.12±0.69)E-03] | 1.36E-03±0.02  [(1.19±2.46)E-03] | 5.72E-03±0.01  [(5.51±1.51)E-03] | 0.01±0.02  [0.01±1.03E-03] | F_(3,600)_=  12.76;  p<0.001 | csf-/pet- < CSF+/PET+  csf-/PET+ < CSF+/PET+  CSF+/pet- < CSF+/PET+  csf-/pet- < CSF+/pet- |
| **N** | 247 | 39 | 46 | 276 | - | - |
| **Annual rate of change of**  **amyloid-β PET SUVr over 2 yr** *(frontal; ΔSUVr/yr)* | 1.30E-03±0.01  [(1.68±0.74)E-03] | 2.57E-03±0.02  [(2.6±2.74)E-03] | 5.24E-03±0.01  [(5.16±1.56)E-03] | 0.01±0.02  [0.01±1.19E-03] | F_(3,600)_=  13.31;  p<0.001 | csf-/pet- < CSF+/PET+  csf-/PET+ < CSF+/PET+ |
| **N** | 247 | 39 | 46 | 276 | - | - |
| **Annual rate of change of**  **amyloid-β PET SUVr over 2 yr** *(cingulate; ΔSUVr/yr)* | 2.44E-03±0.01  [(2.34±0.84)E-03] | 4.79E-03±0.02  [(4.18±3.12)E-03]  *6.2E-03 (-6.3E-03 – 1.72E-02)* | 8.5E-03±0.01  [(8.32±1.66)E-03] | 0.01±0.02  [0.01±1.16E-03] | F_(3,600)_=  8.45;  p<0.001 | csf-/pet- < CSF+/PET+ |
| **N** | 247 | 39 | 46 | 276 | - | - |
| **Annual rate of change of**  **amyloid-β PET SUVr over 2 yr** *(parietal; ΔSUVr/yr)* | 2.72E-03±0.01  [(2.91±0.91)E-03] | -1.58E-03±0.02  [(-1.04±2.91)E-03] | 6.52E-03±0.01  [(6.06±2.04)E-03]  *6.9E-03(-3.9E-03 – 1.3E-02)* | 1.05E-03±0.02  [0.01±1.30E-03]  *1.11E-02(1.25E-04 – 2.25E-02)* | F_(3,600)_=  7.82;  p<0.001 | csf-/pet- < CSF+/PET+  csf-/PET+ < CSF+/PET+ |
| **N** | 247 | 39 | 46 | 276 | - | - |
|  | **csf-/pet-** | **csf-/PET+** | **CSF+/pet-** | **CSF+/PET+** | **Test value;** **p** | **Post-hoc comparison** |
| **Annual rate of change of**  **amyloid-β PET SUVr over 2 yr** *(temporal; ΔSUVr/yr)* | 1.54E-03±0.01  [(1.49±0.87)E-03]  *8E-04 (-6.2E-03 – 7.7E-03)* | -3.31E-04±0.02  [(-0.99±2.71)E-03] | (2.64±9.53)E-03  [(2.49±1.43)E-03] | 8.80E-03±0.02  [(8.95±1.1)E-03]  *9.05E-03(-8.8E-04 – 2.07E-02)* | F_(3,600)_=  9.77;  p<0.001 | csf-/pet- < CSF+/PET+  csf-/PET+ < CSF+/PET+  CSF+/pet- < CSF+/PET+ |
| **N** | 247 | 39 | 46 | 276 | - | - |
| **Annual rate of change of**  **amyloid-β PET SUVr over 4 yr** *(global; ΔSUVr/yr)* | (0.88 ±6.39)E-03  [(8.67±6.04)E-04] | 2.26E-03±0.01  [(2.28±2.39)E-03] | (6.91±8.45)E-03  [(6.7±1.53)E-03] | 9.13E-03±0.01  [(9.19±0.98)E-03] | F_(3,340)_=  15.36; p<0.001 | csf-/pet- < CSF+/PET+  csf-/PET+ < CSF+/PET+  csf-/pet- < CSF+/pet- |
| **N** | 160 | 29 | 30 | 129 | - | - |
| **Annual rate of change of**  **amyloid-β-PET SUVr over 4 yr** *(frontal; ΔSUVr/yr)* | -1.01E-04±0.01  [(0.32±7.13)E-04] | 6.55E-05±0.02  [(0.25±2.91)E-03] | 7.01E-04±0.01  [(6.79±1.82)E-03] | 9.71E-03±0.01  [0.01±1.05E-03] | F_(3,340)_=  17.49;  p<0.001 | csf-/pet- < CSF+/PET+  csf-/PET+ < CSF+/PET+  csf-/pet- < CSF+/pet- |
| **N** | 160 | 29 | 30 | 129 | - | - |
| **Annnual rate of change of**  **amyloid-β PET SUVr over 4 yr** *(cingulate; ΔSUVr/yr)* | (1.48±7.43)E-03  [(1.15±0.72)E-03] | 4.75E-03±0.01  [(4.8±2.71)E-03] | (8.71±9.61)E-03  [(8.4±1.73)E-03] | 8.1E-03±0.01  [(8.58±1.14)E-03]  *8.1E-03(1.7E-03 – 1.66E-02)* | F_(3,340)_=  10.08;  p<0.001 | csf-/pet- < CSF+/PET+  csf-/pet- < CSF+/pet- |
| **N** | 160 | 29 | 30 | 129 | - | - |
| **Annual rate of change of**  **amyloid-β PET SUVr over 4 yr** *(parietal; ΔSUVr/yr )* | (1.55±7.89)E-03  [(1.76±0.73)E-03] | 3.38E-03±0.01  [(3.64±2.74)E-03] | (7.12±9.45)E-03  [(6.85±1.79)E-03] | 0.01±0.01  [0.01±1.14E-03] | F_(3,340)_=  10.4;  p<0.001 | csf-/pet- < CSF+/PET+  csf-/PET+ < CSF+/PET+ |
| **N** | 160 | 29 | 30 | 129 | - | - |
| **Annual rate of change of**  **amyloid-β PET SUVr over 4 yr** *(temporal; ΔSUVr/yr )* | (0.59±7.01)E-03  [(5.14±6.55)E-04] | 8.31E-04±0.01  [(0.42±2.4)E-03] | (4.78±7.46)E-03  [(4.76±1.44)E-03]  *4.7E-03(-3.25E-04 – 8.08E-03)* | 8.54E-03±0.01  [8.73±1.04E-03] | F_(3,340)_=  14.85;  p<0.001 | csf-/pet- < CSF+/PET+  csf-/PET+ < CSF+/PET+ |
| **N** | 160 | 29 | 30 | 129 |  |  |

*Results from univariate ANOVA are corrected for sex, age, diagnostic group and number of APOE-ε4 alleles.*

*Data are reported as mean±standard deviation, unless indicated otherwise.*

*Adjusted estimates of the mean and the respective standard error are reported in square brackets. Sex, age, number of APOE-ε4 alleles and clinical group were entered as nuisance covariates.*

*For groups where variables are non-normally distributed, median (interquartile range) is also reported, in italics.*

*Only significant results (p<0.05 Bonferroni-corrected for multiple comparisons) are reported for post-hoc comparison.*

*Abbreviations: SUVr=standardized uptake value ratio*
