# Supplementary material for: Longitudinal pathways of cerebrospinal fluid and positron emission tomography biomarkers of amyloid-β positivity
Source: Mol Psychiatry. 2020 Dec 11;26(10):5864–74. doi: 10.1038/s41380-020-00950-w (PMC8758501; doi:10.1038/s41380-020-00950-w)
Supplement: Supplementary file 4 — Supplementary Table 3 [file 41380_2020_950_MOESM4_ESM.docx]

**Supplementary Table 3. Baseline and longitudinal measures of tau pathology and neurodegeneration in concordant and discordant biomarker groups**

|  | **csf-/pet-** | **csf-/PET+** | **CSF+/pet-** | **CSF+/PET+** | **Test value;** **p** | **Post-hoc comparison** |
| --- | --- | --- | --- | --- | --- | --- |
| **Baseline CSF p-tau_181_** | 27.94±12.31  [29.87±0.95]  *24.8 (19.3 – 34.58)* | 30.01±12.03  [30.36±2.08]  *28.5*  *(21.33 – 37.08)* | 26.76±12.88  [28.46±1.84] | 58.95±28.15  [52.44±1.32]  *47.9*  *(35.1 – 64.85)* | F_(3,859)_=  53.48; p<0.001 | csf-/pet- < CSF+/PET+  csf-/PET+ < CSF+/PET+  CSF+/pet- < CSF+/PET+ |
| **N** | 300 | 44 | 62 | 461 | - | - |
| **Annual rate of change of CSF p-tau_181_  over 2 yr** | 2.17±7.35  [2.02±0.88]  *0.79 (-1.85 – 4.83)* | 4.39±5.18  [4.76±1.49] | 2.11±5.69  [2.11±1.42] | 4.15±12.68  [4.23±1.1]  *3.08*  *(-1.33 – 11.1)* | F_(3,299)_=  0.94;  p=0.42 | - |
| **N** | 119 | 15 | 19 | 154 | - | - |
| **Baseline CSF t-tau** | 58.37±28.08  [67.58±2.19]  *53 (40.3 – 67.83)* | 66.22±23.38 [70.57±4.15] | 57.17±29.64 [63.31±3.72] | 112.9±57.62 [105.5±2.75] | F_(3,858)_=  37.26;  p<0.001 | csf-/pet- < CSF+/PET+  csf-/PET+ < CSF+/PET+  CSF+/pet- < CSF+/PET+ |
| **N** | 296 | 44 | 61 | 445 | - | - |
| **Annual rate of change of CSF t-tau over 2 yr** | -0.68±7.35  [-1.27±0.83]  *-7.29E-02*  *(-2.52 – 2.36)* | 2.71±5.21  [1.29±1.58] | -0.43±3.72  [-0.19±0.97] | 2.61±14.3  [3.18±1]  *1.96*  *(-1.96 – 6.21)* | F_(3,298)_=  2.56;  p=0.06 | - |
| **N** | 118 | 15 | 19 | 154 | - | - |
| **Baseline MRI Hippocampus/Intracranial volume ratio** | 0.9±1.41  [0.87±0.09]  *5E-03*  *(4.5E-03 – 5.5E-03)* | (5.01±0.82)E-03  [-0.018±0.03] | (4.71±0.81)E-03  [0.03±0.03] | (4.42±0.78)E-03  [0.01±3E-03] | F_(3,791)_=  0.46;  p=0.71 | - |
| **N** | 276 | 42 | 58 | 423 | - | - |
|  | **csf-/pet-** | **csf-/PET+** | **CSF+/pet-** | **CSF+/PET+** | **Test value;** **p** | **Post-hoc comparison** |
| **Annual rate of change of MRI Hippocampus/Intracranial volume ratio over 1 yr** | (-0.77±1.97)E-04  [(-9.1±1.52)E-05] | (-0.59±1.90)E-04  [(-7.48±3.56)E-05] | (-0.56±1.79)E-04  [(-5.61±2.71)  E-05] | (-1.40±2.65)E-04  [(1.29±0.16)  E-04]  *-1E-04*  *(-3E-04 – 0)* | F_(3,605)_=  1.67;  p=0.17 | - |
| **N** | 213 | 29 | 45 | 326 | - | - |
| **Annual rate of change of MRI Hippocampus/Intracranial volume ratio over 1.5 yr** | (-0.95±3.43)E-04  [(-9.94±2.72)E-05]  *-1E-04(-1E-04 – 0)* | (-0.7±1.17) E-04  [(-8.47±2.69)E-05]  *-1E-04*  *(-1.75E-04 – 0)* | (-0.52±1)E-04  [(-4.87±2.25)  E-05] | (-1.33±2.73)E-04  [(-1.29±0.22)  E-04]  *-1E-04*  *(-2E-04 – 0)* | F_(3,420)_=  0.697;  p=0.55 | - |
| **N** | 165 | 20 | 25 | 218 | - | - |
| **Annual rate of change of MRI Hippocampus/Intracranial volume ratio over 2 yr** | (-4.93±9.87)E-05  [(-6.07±0.96)E-05] | (-3.04±6.35)E-05  [(-3.46±1.44)E-05] | (-0.21±1.29)E-04  [(-2.28±2.35)  E-05]  *0(-1E-04 – 0)* | (-1.30±1.32)E-04  [(-1.19±0.13)  E-04]  *-1E-04 (-2E-04 – -1E-04)* | F_(3,327)_=  8.05;  p<0.001 | csf-/pet- < CSF+/PET+  csf-/PET+ < CSF+/PET+  CSF+/pet- < CSF+/PET+ |
| **N** | 134 | 23 | 28 | 150 | - | - |
| **Baseline MRI Whole Brain/Intracranial volume ratio** | 0.70±0.04  [0.70±2.7E-03] | 0.71±0.05  [0.70±6.36E-03]  *0.72 (0.70 – 0.74)* | 0.69±0.05  [0.69±5.37E-03] | 0.69±0.05  [0.69±2.17E-03] | F_(3,773)_=  2.36;  p=0.07 | - |
| N | 270 | 41 | 57 | 413 | - |  |
| **Annual rate of change of MRI Whole Brain /Intracranial volume ratio over 1 yr** | -8.41E-03±0.05  [-0.02±0.01]  *-4.8E-03*  *(-1.1E-02 –*  *7.25E-04)* | -0.07±0.25  [-0.08±0.05]  *-3.5E-03*  *(-9.35E-03 – 1.05E-03)* | -3.86E-03±0.02  [-0.01±0.01]  *-4.1E-03*  *(-1.68E-02 –5.55E-03)* | -0.01±0.18  [-0.01±0.01]  *-8.65-03*  *(-1.20E-02 –*  *-1.13E-03)* | F_(3,606)_=  1.82;  p=0.14 | - |
|  | **csf-/pet-** | **csf-/PET+** | **CSF+/pet-** | **CSF+/PET+** | **Test value;** **p** | **Post-hoc comparison** |
| **N** | 214 | 29 | 45 | 326 | - | - |
| **Annual rate of change of MRI Whole Brain /Intracranial volume ratio over 1.5 yr** | -0.02±0.10  [-0.03±0.01]  *-3.9E-03*  *(-8.15E-03 – 0)* | -0.02±0.08  [-0.03±0.02]  *-3.05E-03*  *(-6.1E-03 –*  *5.25E-04)* | -0.02±0.07  [-0.01±0.02]  *-4.10E-03*  *(-6.65E-03 – 1.70E-03)* | -0.03±0.11  [-0.02±0.01]  *-7.35E-03*  *(-1.42E-02 –*  *-3.05E-03)* | F_(3,421)_=  0.27;  p=0.848 |  |
| **N** | 166 | 20 | 25 | 218 | - | - |
| **Annual rate of change of MRI Whole Brain /Intracranial volume ratio over 2 yr** | -0.02±0.07  [-0.02±0.01]  *-3.4E-03*  *(-6.9E-03 – -3E-04)* | -0.02±0.08  [-0.02±0.02]  *-2.1E-03*  *(-5.7E-03 –*  *-4E-04)* | -0.01±0.06  [-0.01±0.01]  *-2.85E-03*  *(-5.9E-03 –*  *7E-04)* | -0.03±0.08  [-0.03±0.01]  *-7.75E-03*  *(-1.19E-02 –*  *-3.3E-03)* | F_(3,327)_=  0.74;  p=0.53 | - |
| **N** | 134 | 23 | 28 | 150 | - | - |
| **Baseline Alzheimer’s disease-like metabolism [18F]FDG-PET** | 1.31±0.11  [1.28±0.01] | 1.36±0.11  [1.33±0.02] | 1.28±0.13  [1.27±0.02] | 1.19±0.16  [1.21±0.01] | F_(3,849)_=  17.75;  p<0.001 | csf-/pet- < CSF+/PET+  csf-/PET+ < CSF+/PET+  CSF+/pet- < CSF+/PET+ |
| **N** | 298 | 43 | 61 | 455 | - | - |
| **Annual rate of change of Alzheimer’s disease-like metabolism [18F]FDG-PET**  **over 2 yr** | -5.41E-03 ±0.03  [(-8.44±2.94)E-03] | -2.54E-03±0.03  [(-3.74±7.8)E-03] | -0.01±0.03  [-0.01±5.85E-03] | -0.03±0.04  [-0.02±2.71E-03] | F_(3,409)_=  4.88;  p<0.005 | csf-/pet- < CSF+/PET+ |
| **N** | 167 | 20 | 35 | 195 | - | - |

*Results from univariate ANOVA are corrected for sex, age, diagnostic group and number of APOE-ε4 alleles.*

*Data are reported as mean±standard deviation, unless indicated otherwise.*

*Adjusted estimates of the mean and the respective standard error are reported in square brackets. Sex, age, number of APOE-ε4 alleles and clinical group were entered as nuisance covariates.*

*For groups where variables are non-normally distributed, median (interquartile range) is also reported, in italics.*

*Only significant results (p<0.05 Bonferroni-corrected for multiple comparisons) are reported for post-hoc comparison.*

*Abbreviations: [18F]FDG=18F-Fluorodeoxyglucose; p-tau_181_=phosphorylated-tau_181_; t-tau=total-tau*
