# Supplementary material for: Longitudinal pathways of cerebrospinal fluid and positron emission tomography biomarkers of amyloid-β positivity
Source: Mol Psychiatry. 2020 Dec 11;26(10):5864–74. doi: 10.1038/s41380-020-00950-w (PMC8758501; doi:10.1038/s41380-020-00950-w)
Supplement: Supplementary file 5 — Supplementary Table 4 [file 41380_2020_950_MOESM5_ESM.docx]

**Supplementary Table 4. Baseline and longitudinal measures of cognitive and neuropsychiatric deficits in concordant and discordant biomarker groups**

|  | **csf-/pet-** | **csf-/PET+** | **CSF+/pet-** | **CSF+/PET+** | **Test value;** **p-value** | **Post-hoc comparison** |
| --- | --- | --- | --- | --- | --- | --- |
| **Baseline MMSE** | 28.57±1.7  [27.79±0.13]  *29 (28 – 30)* | 28.64±1.82  [27.94±0.29]  *29 (28 – 30)* | 28.18±2.18  [27.66±0.27] | 26.59±3.06  [27.23±0.12] | F_(3,859)_=3.51; p<0.05 | csf-/pet- < CSF+/PET+ |
| **N** | 300 | 44 | 62 | 461 | - | - |
| **Annual rate of change of MMSE over 1 yr** | -0.06±1.65  [-0.14±0.13]  *0 (-0.89 – 0.88)* | -0.01±1.41  [-0.01±0.27] | -0.31±1.57  [-0.43±0.2] | -1±2.32  [-0.93±0.13]  *-0.87(-1.92 – 0)* | F_(3,703)_=6.08;  p<0.001 | csf-/pet- < CSF+/PET+ |
| **N** | 252 | 32 | 54 | 373 | - | - |
| **Annual rate of change of MMSE over 2 yr** | -0.06±0.82  [-0.16±0.06] | 1.5E-04±0.69  [-0.06±0.11]  *0 (-0.48 – 0.45)* | -0.40±1.03  [-0.47±0.15]  *0 (-0.48 – 0.45)* | -0.68±1.24  [-0.57±0.07] | F_(3,657)_=6.43;  p<0.001 | csf-/pet- < CSF+/PET+  csf-/PET+ < CSF+/PET+ |
| **N** | 265 | 40 | 51 | 309 | - | - |
| **Annual rate of change of MMSE over 3.5 yr** | -0.04±0.49  [-0.11±0.05] | 0.04±0.36  [-0.03±0.06]  *0(-0.24 – 0.24)* | -0.11±0.46  [-0.13±0.08] | -0.69±1.21  [-0.59±0.08]  *-0.33(-0.98 – 0)* | F_(3,526)_=10.18;  p<0.001 | csf-/pet- < CSF+/PET+  csf-/PET+ < CSF+/PET+  CSF+/pet- < CSF+/PET+ |
| **N** | 222 | 38 | 41 | 233 | - | - |
| **Baseline ADAS-cog11** | 9.47±6.25  [9.02±0.42]  *8 (6 – 11)* | 9.68±6.82  [9.47±1.02]  *7.5 (5 – 12.75)* | 10.32±7.27  [10.15±0.92]  *8 (5 – 13)* | 10.43±7.23  [10.77±0.38] | F_(3,859)_=2.79; p<0.05 | csf-/pet- < CSF+/PET+ |
| N | 300 | 44 | 62 | 461 | - | - |
| **Annual rate of change of ADAS-cog11 over 1 yr** | 0.24±3.58  [0.31±0.24]  *0 (-1.97 – 2.01)* | 2.27±10.77  [2.3±1.8]  *0 (-1.02 – 1.12)* | 0.49.±3.2  [0.48±0.43] | 0.74±4.43  [0.68±0.26] | F_(3,716)_=2.12;  p=0.1 | - |
|  | **csf-/pet-** | **csf-/PET+** | **CSF+/pet-** | **CSF+/PET+** | **Test value;** **p-value** | **Post-hoc comparison** |
| **N** | 277 | 35 | 56 | 356 | - | - |
| **Annual rate of change of ADAS-cog11 over 2 yr** | 0.39±2.32  [0.42±0.15]  *0 (-1 – 1.02)* | 0.58±2.01  [0.65±0.37]  *0 (-0.5 – 1.25)* | 0.48.±2.34  [0.40±0.33]  *0.3*  *(-0.97 – 1.51)* | 0.56±2.22  [0.54±0.14]  *0.04*  *(-0.99 – 1.49)* | F_(3,649)_=0.16;  p=0.92 | - |
| **N** | 256 | 29 | 50 | 322 | - | - |
| **Annual rate of change of ADAS-cog11 over 3.5 yr** | 0.52±1.77  [0.54±0.14] | 0.52±1.17  [0.56±0.24] | 0.5±1.51  [0.44±0.22] | 0.72±1.89  [0.71±0.15] | F_(3,520)_=0.38;  p=0.77 | - |
| **N** | 207 | 23 | 43 | 255 | - | - |
| **Baseline RAVLT** | 4.9±2.63  [4.98±0.17] | 4.77±3.17  [4.79±0.41] | 4.47±2.28  [4.55±0.35] | 4.4±2.76  [4.34±0.14] | F_(3,858)_=2.37; p=0.07 | - |
| **N** | 299 | 44 | 62 | 461 | - | - |
| **Annual rate of change of RAVLT** **over 1 yr** | -0.1±2.37  [-0.19±0.16] | -0.41±2.6  [-0.44±0.41] | -0.12±2.28  [-0.21±0.32] | 4.42E-03±2.37  [0.09±0.14] | F_(3,714)_=0.84;  p=0.47 | - |
| **N** | 275 | 34 | 56 | 357 | - | - |
| **Annual rate of change of RAVLT** **over 2 yr** | -0.18±1.18  [-0.22±0.09] | -0.22±1.17  [-0.22±0.23] | 0.9±1.13  [0.06±0.18] | -0.16±1.28  [-0.13±0.08] | F_(3,649)_=0.74;  p=0.53 | - |
| **N** | 252 | 29 | 50 | 236 | - | - |
| **Annual rate of change of RAVLT** **over 3.5 yr** | -0.2±0.84  [-0.23±0.07] | -0.09±0.98  [-0.1±0.18]  *-0.19*  *(-0.65 – 0.25)* | -0.2±0.67  [-0.22±0.13] | -0.19±0.85  [-0.15±0.06] | F_(3,512)_=0.34;  p=0.8 | - |
| **N** | 207 | 23 | 43 | 247 | - | - |
| **Baseline GDS** | 1.48±1.7  [1.44±0.1]  *1 (0 – 2)* | 2.09±2.07  [2.13±0.3] | 1.5±1.56  [1.4±0.2] | 1.44±1.36  [1.47±0.07] | F_(3,857)_=2.75;  p<0.05 | csf-/pet- < csf-/PET+  CSF+/PET+ < csf-/PET+ |
| **N** | 299 | 44 | 62 | 460 |  |  |

*Results from univariate ANOVA are corrected for sex, age, diagnostic group and number of APOE-ε4 alleles.*

*Data are reported as mean±standard deviation, unless indicated otherwise.*

*Adjusted estimates of the mean and the respective standard error are reported in square brackets. Sex, age, number of APOE-ε4 alleles and clinical group were entered as nuisance covariates.*

*For groups where variables are non-normally distributed, median (interquartile range) is also reported, in italics.*

*Only significant results (p<0.05 Bonferroni-corrected for multiple comparisons) are reported for post-hoc comparison.*

*Abbreviations: ADAS-cog11= Alzheimer’s disease Assessment Scale-cognitive subscale; GDS=Geriatric Depression Scale; MMSE=Mini Mental State Examination; RAVLT=Rey Auditory Verbal Learning Test, learning subscal*
