# Supplementary material for: Longitudinal pathways of cerebrospinal fluid and positron emission tomography biomarkers of amyloid-β positivity
Source: Mol Psychiatry. 2020 Dec 11;26(10):5864–74. doi: 10.1038/s41380-020-00950-w (PMC8758501; doi:10.1038/s41380-020-00950-w)
Supplement: Supplementary file 6 — Supplementary Table 5 [file 41380_2020_950_MOESM6_ESM.docx]

**Supplementary Table 5. Evaluation of confounding factors in concordant and discordant biomarker groups**

|  | **csf-/pet-** | **csf-/PET+** | **CSF+/pet-** | **CSF+/PET+** | **Test value;** **p-value** | **Post-hoc comparison** |
| --- | --- | --- | --- | --- | --- | --- |
| **Ventricles/Intracranial Volume Ratio at MRI** | 0.02±0.01  [0.02±1E-03]  *0.02 (0.01 – 0.03)* | 0.02±0.02  [0.02±3E-03]  *0.02 (0.01 – 0.02)* | 0.03±0.02  [0.03±3E-03] | 0.03±0.01  [0.03±1E-03] | F_(3,673)_=8.66; p<0.001 | csf-/pet- < CSF+/PET+  CSF+/pet- < CSF+/PET+ |
| **N** | 236 | 36 | 50 | 359 | - | - |
| **Frequency/severity of sleep disturbances (NPI-K Total Score)** | 0.7±1.85  [0.67±0.12]  *0 (0 – 0)* | 0.84±1.68  [0.81±0.26]  *0 (0 – 1)* | 0.6±1.81  [0.58±0.24]  *0 (0 – 0)* | 0.74±1.87  [0.77±0.09]  *0 (0 – 0)* | F_(3,852)_=0.26; p=0.86 | - |
| **N** | 294 | 44 | 62 | 460 | - | - |
| **Time interval between PET tracer injection and image acquisition, minutes** | 50.18±3.79  [50.1±0.22]  *50 (49.95 – 50.02)* | 51.22±3.87  [51.19±0.58]  *50.0 3(50 – 51.1)* | 50.09±1.51  [50.08±0.2]  *50 (49.21 – 50.82)* | 50.4±3.64  [50.46±0.21]  *50 (49.98 – 50.6)* | F_(3,857)_=1.4;  p=0.24 | - |
| **N** | 300 | 44 | 62 | 459 | - | - |
| **Presence of infarcts, N (%)** | 14 (4.7%) | 3 (6.8%) | 5 (8.1%) | 18 (3.9%) | χ^2^_(3)_=2.69;  P=0.44 | - |
| **N** | 300 | 44 | 62 | 461 | - | - |
| **Volume of white matter hyper-intensities at MRI [adjusted for total intracranial volume]** | (4.7±6.27)E-03  [(4.82±0.39)E-03]  *[2.73(1.3 – 5.53)]*  *E-03* | (5.75±8.4)E-03  [(5.95±1.43)E-03]  *[3.3(1 – 6.1)]*  *E-03* | (5.22±8.95)E-03  [(5.21±1.27)E-03]  *[2.5(1.15 – 6.43)]*  *E-03* | (4.69±5.7)E-03  [(4.6±0.29)E-03]  *[2.5(1.1 – 5.6)]*  *E-03* | F_(3,808)_=0.57; p=0.63 | - |
| **N** | 274 | 37 | 54 | 451 | - | - |
| **CSF amyloid-β_40_, pg/mL** | 8718.85±2471.31  [8757.16±157.56] | 9788.26±2336.69 [9707.42±369.84] | 7369.64±2846.61  [7410.66±314.37] | 8247.62±2343.2  [8228.87±125.7] | F_(3,814)_=9.3; p<0.001 | CSF+/pet- < csf-/pet- CSF+/PET+ < CSF+/pet- |
| **N** | 283 | 43 | 59 | 437 | - | - |
|  | **csf-/pet-** | **csf-/PET+** | **CSF+/pet-** | **CSF+/PET+** | **Test value;** **p-value** | **Post-hoc comparison** |
| **Baseline amyloid-β PET SUVr** *(occipital)* | 0.76±0.06  [0.76±0.01] | 0.83±0.06  [0.83±0.01] | 0.78±0.06  [0.79±0.01] | 0.94±0.13 [0.93±0.01] | F_(3,859)_=  125.1;  p<0.001 | csf-/pet- < CSF+/PET+  csf-/PET+ < CSF+/PET+  CSF+/pet- < CSF+/PET+  csf-/pet- < csf-/PET+  csf-/pet- < CSF+/pet-  CSF+/pet- < csf-/PET+ |
| **N** | 300 | 44 | 62 | 461 | - | - |

*Results from univariate ANOVA are corrected for sex, age, diagnostic group and number of APOE-ε4 alleles.*

*Data are reported as mean±standard deviation, unless indicated otherwise.*

*Adjusted estimates of the mean and the respective standard error are reported in square brackets. Sex, age, number of APOE-ε4 alleles and clinical group were entered as nuisance covariates.*

*For groups where variables are non-normally distributed, median (interquartile range) is also reported, in italics.*

*Only significant results (p<0.05 Bonferroni-corrected for multiple comparisons) are reported for post-hoc comparison.*

*Abbreviations: NPI-K=Neuropsychiatric Inventory, sleep subscale; SUVr= standardized uptake value ratio*
